# Tetraarylpyrrolo[3,2-*b*]pyrrole-BODIPY dyad: a molecular rotor for FRET-based viscosity sensing

**DOI:** 10.3389/fchem.2024.1473769

**Published:** 2024-10-10

**Authors:** Richa Agrawal, Sudip Gorai, Sunil Suresh Yadav, Amey P. Wadawale, Soumyaditya Mula

**Affiliations:** ^1^ Bio-Organic Division, Bhabha Atomic Research Centre, Mumbai, India; ^2^ Homi Bhabha National Institute, Mumbai, India; ^3^ National Centre for Nanoscience and Nanotechnology, University of Mumbai, Mumbai, India; ^4^ Chemistry Division, Bhabha Atomic Research Centre, Mumbai, India

**Keywords:** tetraarylpyrrolo[3,2-*b*]pyrrole, BODIPY, dyad, viscosity sensor, molecular rotor, FRET

## Abstract

With the aim to develop a FRET-based viscosity sensor, two dyad molecules, **4** and **5**, comprising tetraarylpyrrolo[3,2-*b*]pyrrole (TAPP) (donor) and naked boron-dipyrromethene (BODIPY) dyes (acceptor), were designed. Dyads were synthesized via acid-catalyzed multicomponent reactions followed by Sonogashira coupling. In both dyads, the BODIPY and TAPP moieties are linked through phenylethynyl groups, which allow free rotation of the BODIPY dyes; that is, they can act as molecular rotors. This was supported by X-ray crystallographic and DFT-optimized structures. Spectroscopic studies also confirmed the presence of both TAPP and BODIPY dyes in dyads with no electronic interactions that are suitable for fluorescence resonance energy transfer (FRET). Very high energy transfer efficiency (ETE >99%) from the donor TAPP moiety to the acceptor BODIPY moiety on excitation at the TAPP part was observed. However, due to the non-fluorescent nature of naked BODIPY dyes, no fluorescence emission was observed from the BODIPY moiety in both dyads. With increasing solvent viscosities, emission from the BODIPY moieties increases due to the restricted rotation of the BODIPY moieties. Plotting the logarithms of the fluorescent intensity of dyad **5** and the viscosity of the solution showed a good linear correlation obeying a Förster–Hoffmann equation. Non-fluorescent dyad **5** in methanol became greenish-yellow fluorescent in a methanol/glycerol (1:1) solvent. Furthermore, with an increase in the temperature of the methanol/glycerol (1:1) system, as the viscosity decreases, the fluorescence also starts decreasing. Thus, dyad **5** is capable of sensing the viscosity of the medium via a FRET-based “Off-On” mechanism. This type of viscosity sensor with a very large pseudo-Stokes shift and increased sensitivity will be useful for advancing chemo-bio sensing and imaging applications.

## Graphical Abstract

**Figure:**
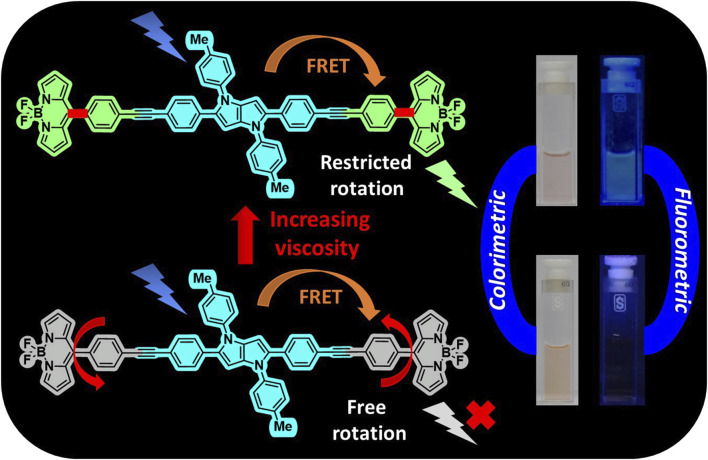
TOC.

## Introduction

Molecular dyads with a combination of a donor chromophore (absorbs at lower wavelengths) and an acceptor molecule (absorbs at higher wavelengths) have diverse applications in different fields ranging from artificial light-harvesting to advanced biotechnology (Altan Bozdemir et al., 2011; Odobel et al., 2013). A dyad with suitably matched optical properties of the donor and acceptor can act as a fluorescence resonance energy transfer (FRET) system. In this type of system, photoexcitation of the donor will lead to the transfer of its excitation energy to the acceptor, showing emission from the acceptor molecule at a higher wavelength (Lin et al., 2010). The difference between the wavelengths of emission maximum of the acceptor and the absorption maximum of the donor is a pseudo-Stokes shift, which is higher than a Stokes shift of the acceptor. These kinds of systems are important for energy tunneling in artificial light-harvesting antennas. In addition, the enhanced pseudo-Stokes shifts of these dyads are highly useful for developing chemical sensors (Guliyev et al., 2009; Qu et al., 2012) and advanced bio-imaging agents (Ulrich et al., 2005; Wu et al., 2009; Wu et al., 2020).

Viscosity is an important cellular parameter used to control the intracellular chemical signaling interactions of biomolecules and the diffusion of active metabolites within cellular systems. Intracellular viscosity changes are indicative of different diseases, such as hypertension, diabetes (Nadiv et al., 1994), atherosclerosis (Deliconstantinos et al., 1995), and Alzheimer’s disease (Zubenko et al., 1999). Consequently, finding novel techniques that could image subcellular viscosity in order to recognize problems linked to viscosity is immensely important. Thus, various viscosity sensors have been developed over the years (Su et al., 2017; Lee et al., 2018; Miao et al., 2019; Ma et al., 2020). The majority of these are fluorescence “Off-On” or “On-Off” types, and the accuracy of these sensors is limited by the self-absorption of the fluorophores. In that respect, the FRET-based system has a potential advantage because its large pseudo-Stokes shift will nullify the effect of self-absorption, and viscosity measurement depends on the relative orientation of the two independent chromophores where one (acceptor) molecule is sensitive and other (donor) is less sensitive towards viscosity response. Designing this type of FRET-based viscosity sensor requires a molecular pair as a dyad where the energy donor and acceptor are connected via a linker with an optimal distance.

Boron-dipyrromethene (BODIPY) dyes have emerged as a highly important class of dyes for numerous applications (Loudet and Burgess, 2007; Mula et al., 2008; Choudhary et al., 2024; Mula, 2024). This is because of their exceptional properties, like high molar absorptivity, high fluorescence, high photostability, and relatively easy customization (Loudet and Burgess, 2007; Ulrich et al., 2008). However, small Stokes shifts restrict their wide applicability in different applications, including chemo/biosensing and imaging. The majority of the chemo/bio-sensors and imaging agents developed using BODIPY dyes are “Off-On” or “On-Off” types (Boens et al., 2012; Gorai et al., 2022; Choudhary and Mula, 2023). A small Stokes shift makes the detection problematic in this kind of sensor mainly due to the self-absorption of the fluorescence light. Suitably designed dyads with large pseudo-Stokes shifts can be used for sensing purposes with enhanced sensitivity. Thus, the development of dyads with enhanced pseudo-Stokes shift along with high energy transfer efficiency (ETE) will be ideal for advanced sensing and imaging applications.

In the past, various BODIPY-based dyads were synthesized for solar energy harvesting purposes. In all these cases, BODIPYs have been extensively used both as energy donors and energy acceptors (Harriman et al., 2009; Bozdemir et al., 2010; Mula et al., 2010). Typically, BODIPYs have absorption in the green region (∼500 nm), and tuning it to the blue region is hardly possible (Hee Kim and Kim, 2019). Thus, very often, polycyclic aromatic hydrocarbons (PAHs) like pyrene, perylene, anthracene, triptycene, fluorene, etc., are attached to the BODIPY core as blue energy donors. These donors absorb in the blue region (200–400 nm) and efficiently transfer their excitation energy to the BODIPY core, enhancing the pseudo-Stokes shifts of BODIPY dyes (Ulrich et al., 2005; Iehl et al., 2012; Porcu et al., 2022). These types of dyads could be highly useful as sensing/imaging agents. From a synthetic point of view, these PAHs are difficult to synthesize or functionalize. The development of custom-made dyads based on a PAH-BODIPY frame is always challenging. Thus, introducing new fluorophores with absorption, emission in the blue region along with easy synthesis and functionalization procedures will be a real game changer.

**CHART 1:**
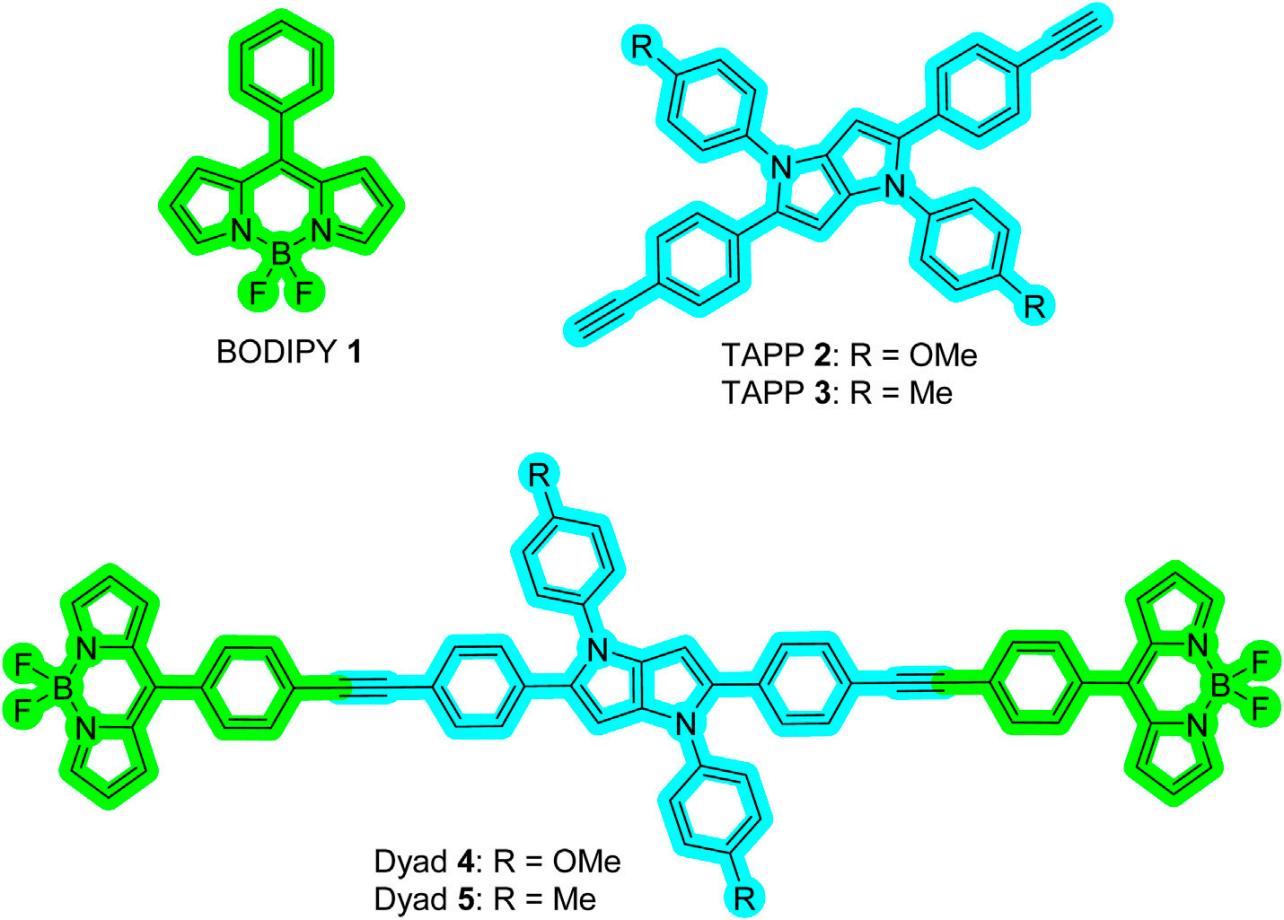
Chemical structures of BODIPY **1** (acceptor), TAPP **2**, **3** (donor), and Dyads **4**, **5**.

A new class of fluorescent dye, namely, tetraarylpyrrolo[3,2-*b*]pyrrole (TAPP) (Chart 1), has been known since 2013 (Janiga et al., 2013; Krzeszewski et al., 2017). Recent developments showed that synthesizing these dyes is relatively easy compared to other PAHs, and importantly, there are ample scopes for post-functionalization of the TAPP core to synthesize advanced chromophores for diverse applications (Janiga et al., 2014; Liu et al., 2017; Krzeszewski et al., 2018). These dyes are being used for photochromic analysis of halocarbons, direct solvent probing via H-bonding interactions, two-photon absorption application, aggregation-induced emission, and development of resistive memory devices, MOFs, organic opto-electronics, and organic light-emitting diodes (OLEDs) (Wu et al., 2016; Dereka and Vauthey, 2017; Ji et al., 2017; Krzeszewski et al., 2017; Li et al., 2017; Hawes et al., 2018; Wang et al., 2018). TAPP has strong absorption at ∼400 nm and high blue emission (∼450 nm) (Tasior et al., 2020). Superior photophysical properties and easy synthesis prompted us to use these dyes as blue energy donors in molecular dyads. As the TAPP molecules are blue energy donors, we have taken the naked BODIPY **1** (Chart 1) dye as an acceptor because (i) its absorption profile has reasonable overlap with the emission profile of TAPP and (ii) it shows a molecular rotor property (Chakraborty et al., 2021). Due to the molecular rotor property, naked BODIPY dyes are being used as viscosity sensors, where these dyes showed “Off-On” fluorescence sensing with an increase in viscosity of the medium (Sunahara et al., 2007). A narrow Stokes shift remains a problem for these sensors in accurately detecting analytes. In this respect, viscosity detection is anticipated to be much easier with enhanced sensitivity if the naked BODIPY dye can be used in a dyad-based molecular rotor system. Thus, we have designed two TAPP-BODIPY-based dyads where TAPP and naked BODIPY dyes are linked through the C2/C5 of the TAPP moiety using a phenylethynyl linker. This linker will allow these two chromophores to remain electronically separated, allowing the free rotations of the chromophores (Chart 1). These dyads are expected to be non-fluorescent and to show enhanced fluorescence with increasing viscosity. In this report, the synthesis and characterization of two TAPP-BODIPY dyads are reported. Their photophysical properties and resonance energy transfer were investigated, and they showed highly efficient energy transfer from the TAPP to the BODIPY moiety. Finally, the potential application of these newly synthesized dyads as viscosity sensors is discussed.

## Results and discussion

### Synthesis

**SCHEME 1:**
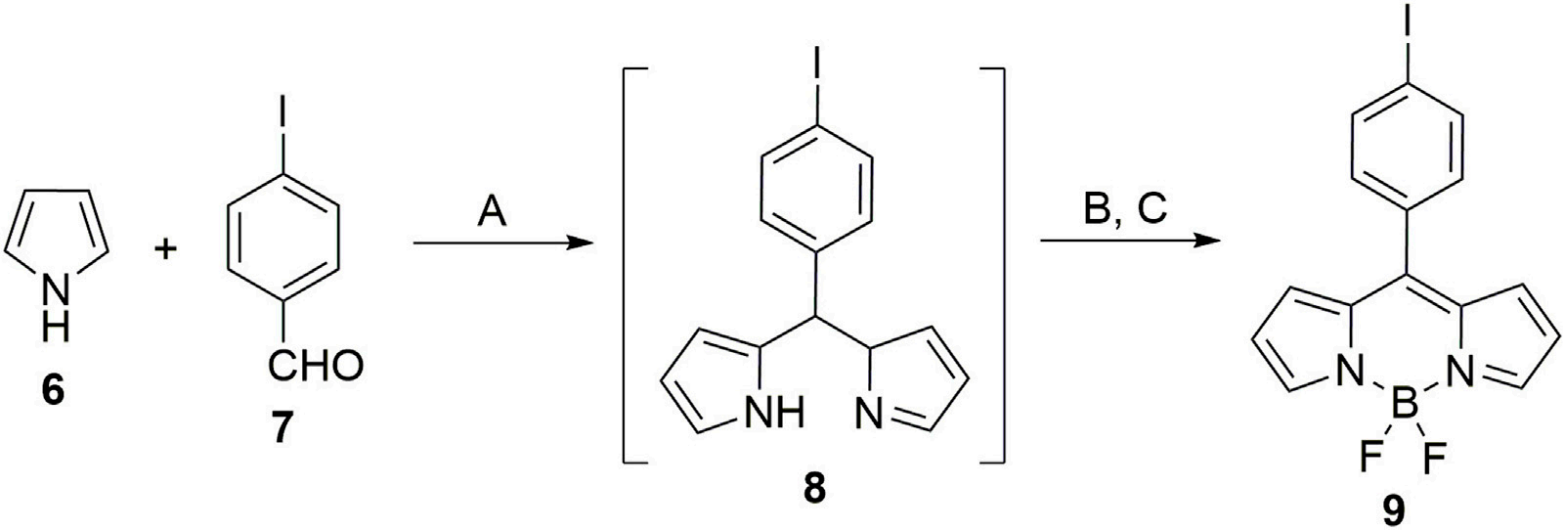
Synthesis of acceptor BODIPY **9**: Reaction conditions: **(A)** TFA (cat. amount), dry DCM, 25°C, 12 h; **(B)** DDQ, 25°C, 4 h; **(C)** NEt_3_, BF_3_. Et_2_O, 25°C, 12 h.

Initially, the acceptor, BODIPY **9,** was synthesized. For the synthesis of BODIPY **9**, pyrrole (**6**) was condensed with *p*-iodo benzaldehyde (**7**) in the presence of a catalytic amount of acid to form corresponding dipyrromethane (**8**). DDQ was used to oxidize **8** followed by its complexation with BF_3_. OEt_2_ furnished the acceptor BODIPY **9** (Scheme 1).

**SCHEME 2:**
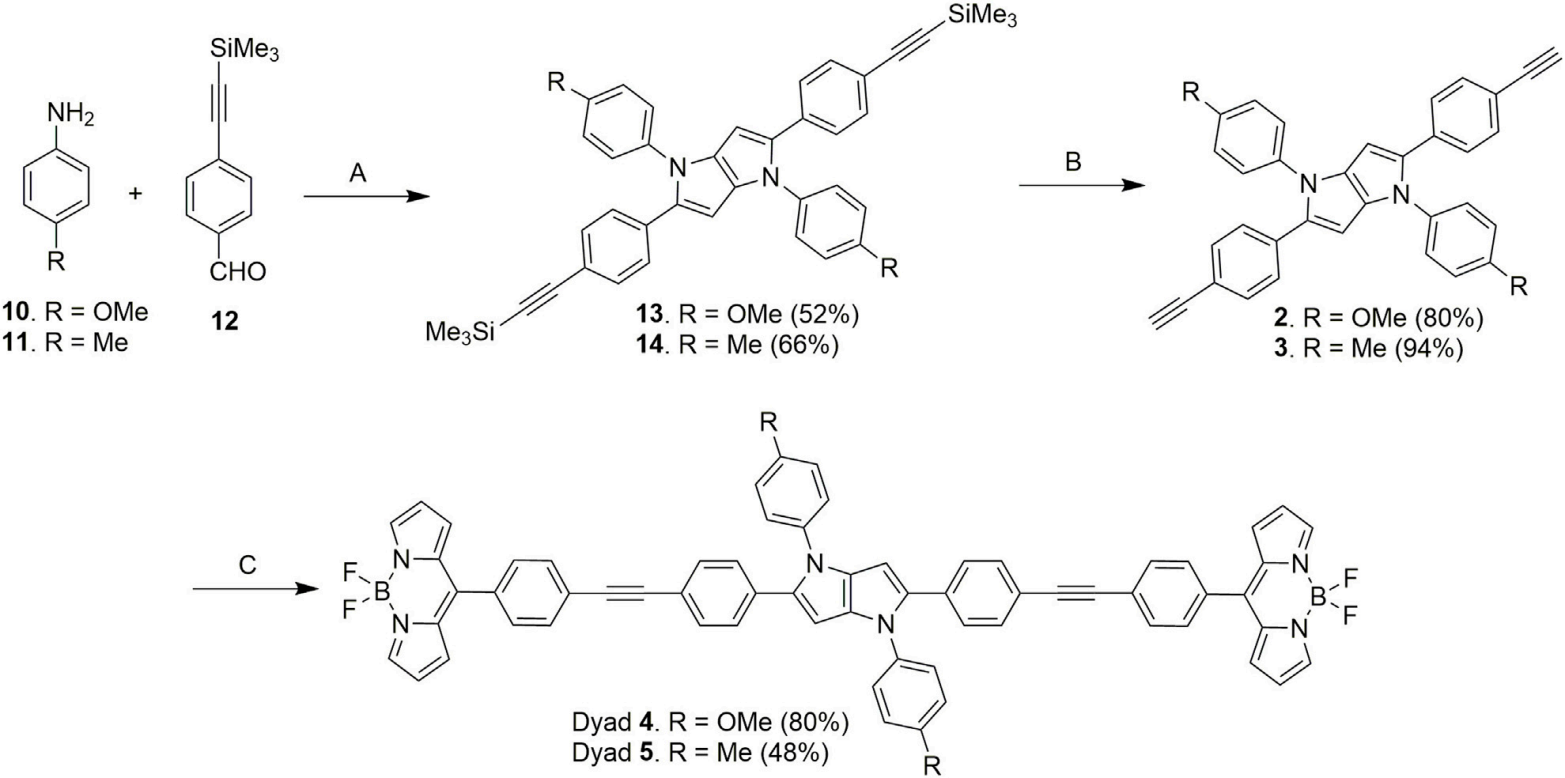
Synthesis of TAPP **2**, **3** and TAPP-BODIPY dyads **4**, **5**. Reaction conditions: **(A)** (i) AcOH: toluene, 50°C, 1 h; (ii) 2,3-butanedione, Fe(ClO_4_)_3_.H_2_O (6 mol%), 50°C, 12 h; **(B)** K_2_CO_3_, MeOH, 25°C, 6 h; **(C)** BODIPY **9**, DIPA, *trans*-Pd(PPh_3_)_2_Cl_2_ (10 mol%), CuI (5 mol%), 25°C, dry THF, 12 h.

**FIGURE 1:**
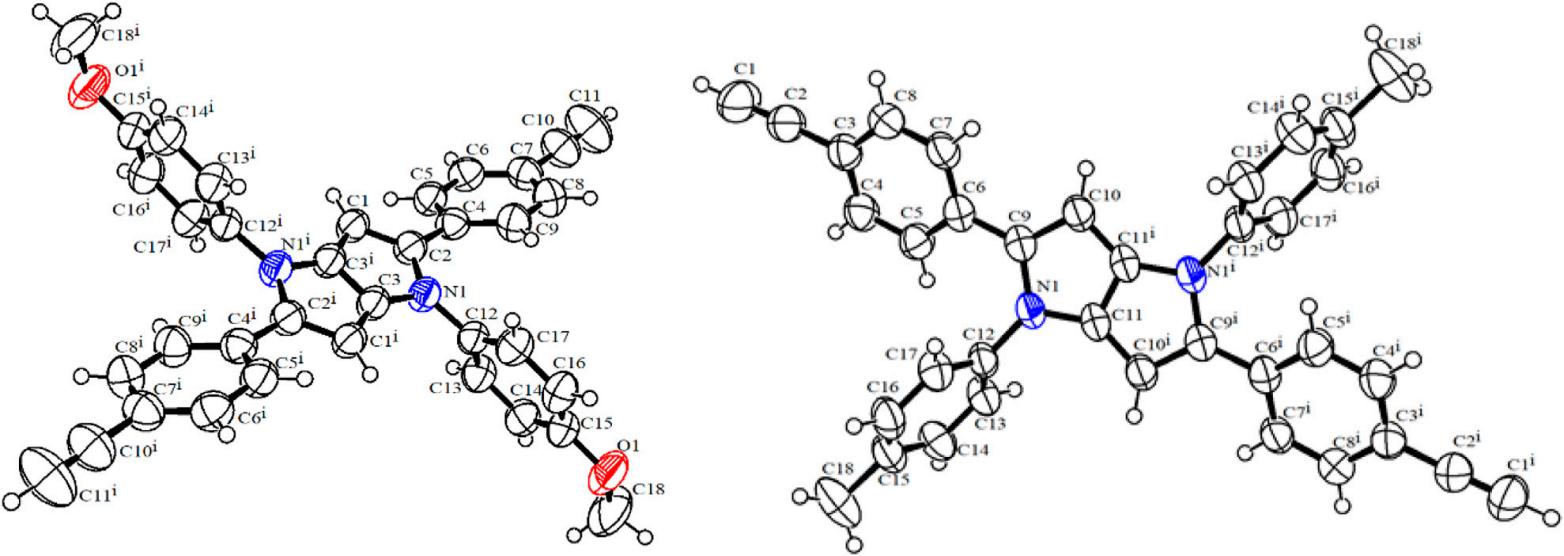
X-ray crystal structures of TAPP **2** and **3**.

Next, the donor molecules TAPP **2** and **3** were synthesized via multicomponent condensation reactions. The acid-catalyzed reaction of *p*-methoxyaniline (**10**) and 4-((trimethylsilyl)ethynyl)benzaldehyde (**12)** formed the corresponding Schiff base, which was simultaneously condensed with 2,3-butanedione using Fe(ClO_4_)_3_.H_2_O as the catalyst to furnish TAPP **13**. In another effort, *p*-methylaniline (**11**) and 4-((trimethylsilyl)ethynyl)benzaldehyde (**12**) were condensed in an acid-catalyzed reaction to generate the Schiff base, which was further condensed with 2,3-butanedione to furnish TAPP **14** in good yield (Tasior et al., 2020). Then, both TAPP **13** and **14** were desilylated to obtain the corresponding TAPP **2** and **3**, respectively (Scheme 2). All the dyes were characterized by NMR spectroscopy and mass spectrometric analyses. For example, in ^1^H NMR spectrum of **13**, phenyl protons were resonated as four doublets of four proton integration each in the aromatic region. Characteristic singlets for C-3/6 protons of the TAPP moiety and the methoxy and trimethyl silyl groups were observed at 6.34 ppm, 3.83 ppm, and 0.23 ppm, respectively, which confirmed the structure of **13** (Supplementary Figure S5). The disappearance of a singlet for the trimethylsilyl groups at 0.23 ppm and the appearance of a singlet at 3.08 ppm for the acetylenic protons confirm the formation of TAPP **3** (Supplementary Figure S11). Finally, the structures of TAPP **2** and **3** were confirmed by single-crystal X-ray crystallographic studies, as shown in Figure 1. Crystal structures of TAPP **2** and **3** showed that the pyrrolo[3,2-*b*]pyrrole units are planar, but the four aryl units remain out of plane, making them electronically non-conjugated with the pyrrolo[3,2-*b*]pyrrole units.

**FIGURE 2:**
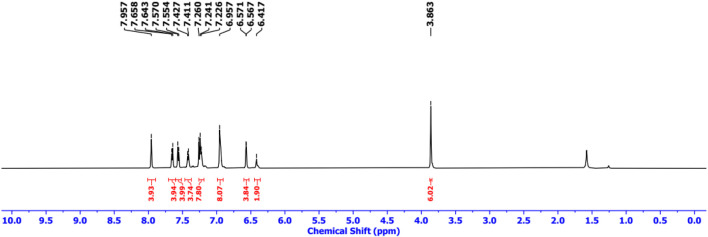
^1^H NMR (500 MHz) spectrum of dyad **4** in CDCl_3_.

Finally, Sonogashira coupling of BODIPY **9** with TAPP **2** and **3** separately furnished dyads **4** and **5,** respectively (Scheme 2). The structures of both dyads were confirmed by NMR spectroscopy and mass spectrometry analyses. In the ^1^H NMR spectrum of dyad **4**, all the characteristic peaks for both the TAPP **2** and BODIPY **9** moieties were present (Figure 2; Supplementary Figure S15). The acetylenic proton signal of the TAPP **2** moiety was absent, which confirmed the coupling of the two moieties through the acetylenic bond.

### DFT calculations

Orientations of the donors and acceptors in the dyads are important for efficient energy transfer between them. Thus, DFT studies were done to optimize the ground state geometries of the dyads **4** and **5** using the B3LYP/6-31G level of theory. These are shown in Table 1; Supplementary Table S4. In both dyads, the pyrrolo-pyrrole units are planar as expected, and the four attached aryl rings remain twisted with respect to the pyrrolo-pyrrole plane. Similar observations were found from the X-ray crystallographic structures discussed *vide supra*. Furthermore, BODIPY moieties are planar, but the attached 8-phenyl groups are twisted with respect to the BODIPY core. Thus, as a whole, in both dyads, the donor TAPP moieties and the acceptor BODIPYs are twisted with respect to each other; that is, they are not electronically conjugated. This suggests that energy transfer is possible in both dyads **4** and **5**. The HOMO-LUMO structures and energies of both dyads are also calculated and tabulated in Table 1; Supplementary Table S4. None of the FMO’s electron densities are distributed over both chromophores. The electron densities in the HOMOs of both dyads are located on the pyrrolo-pyrrole unit and shifted towards the BODIPY core in LUMOs. These also indicate that the TAPP and BODIPY moieties are not conjugated; thus, energy transfer is feasible in both dyads, as discussed below.

**TABLE 1:** Ground-state optimized structures and calculated HOMO and LUMO surfaces of dyads **4** and **5**.

Dyad	Optimized structure	HOMO (eV)	LUMO (eV)
4	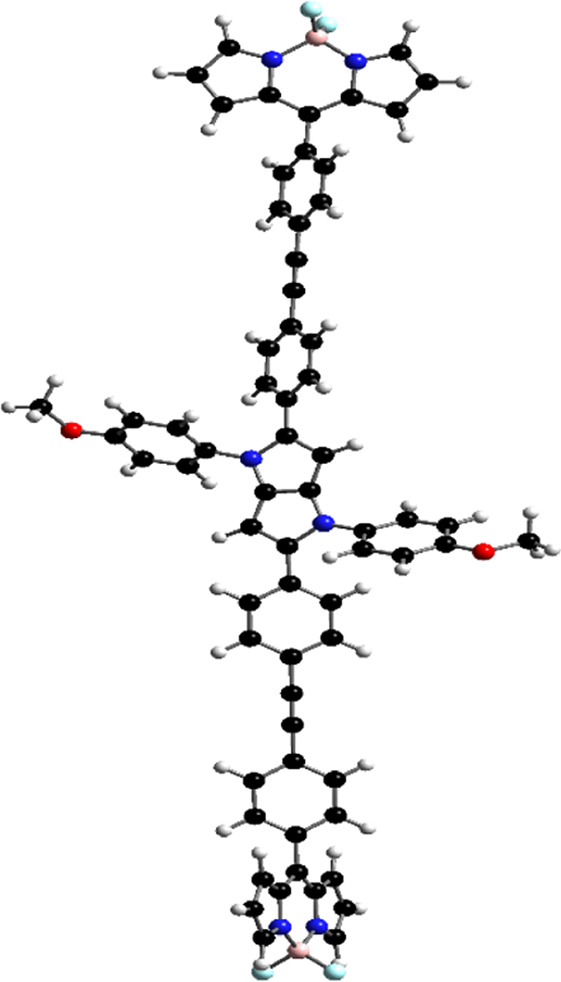	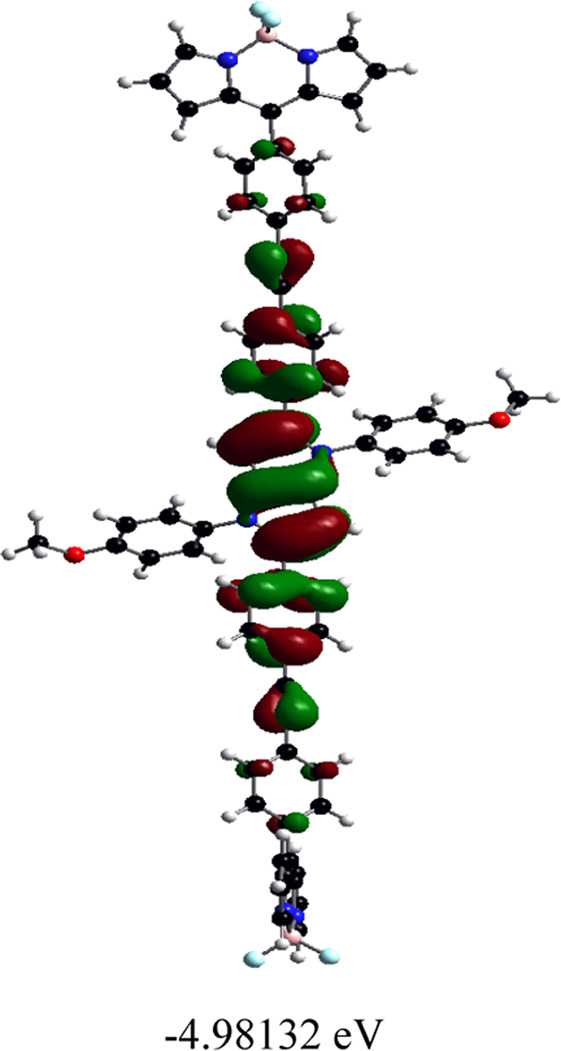	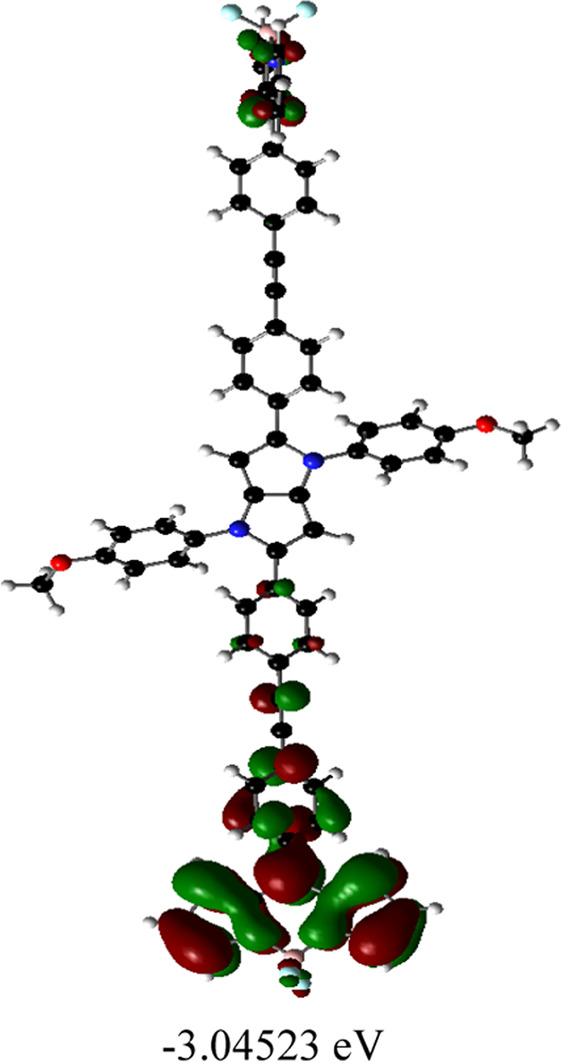
5	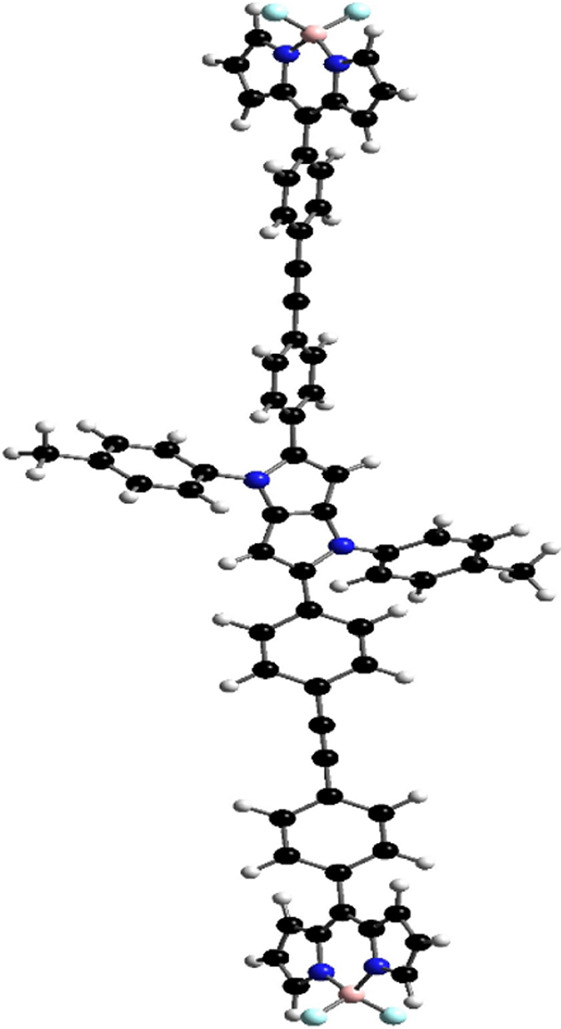	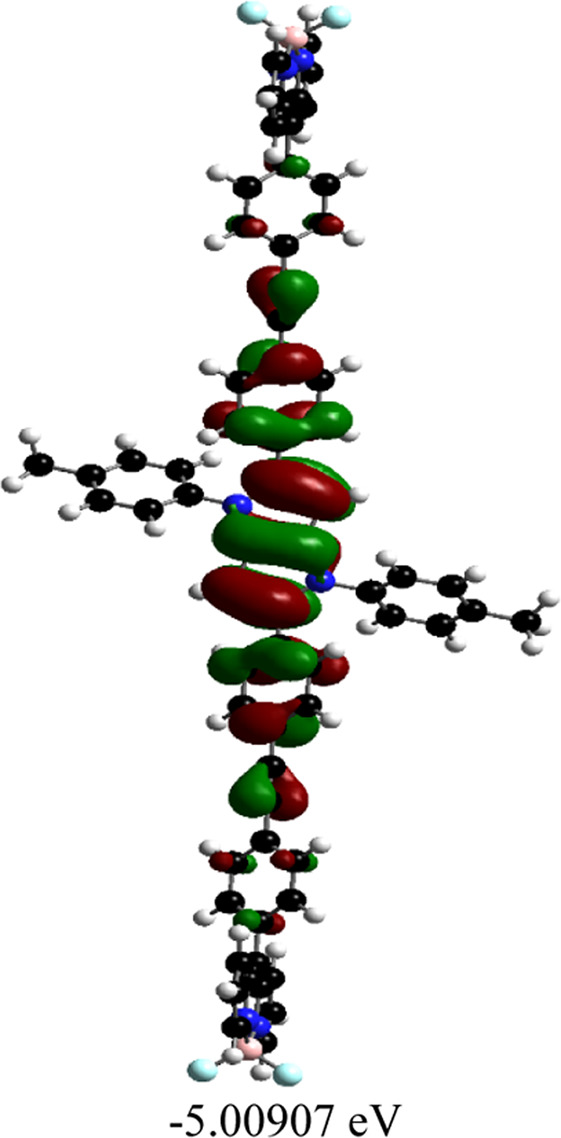	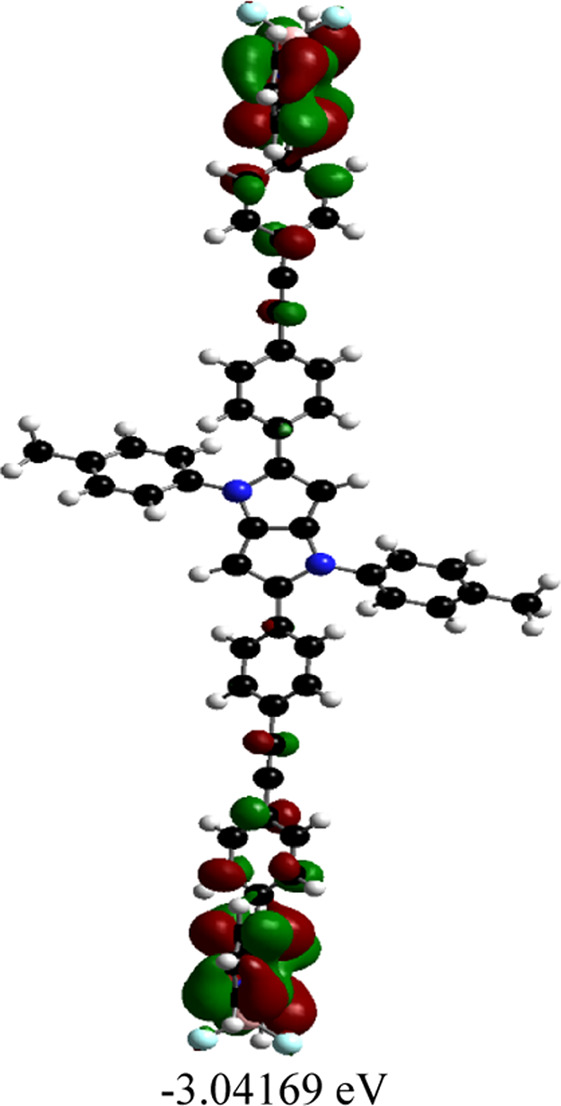

### Steady-state photophysical study

**FIGURE 3:**
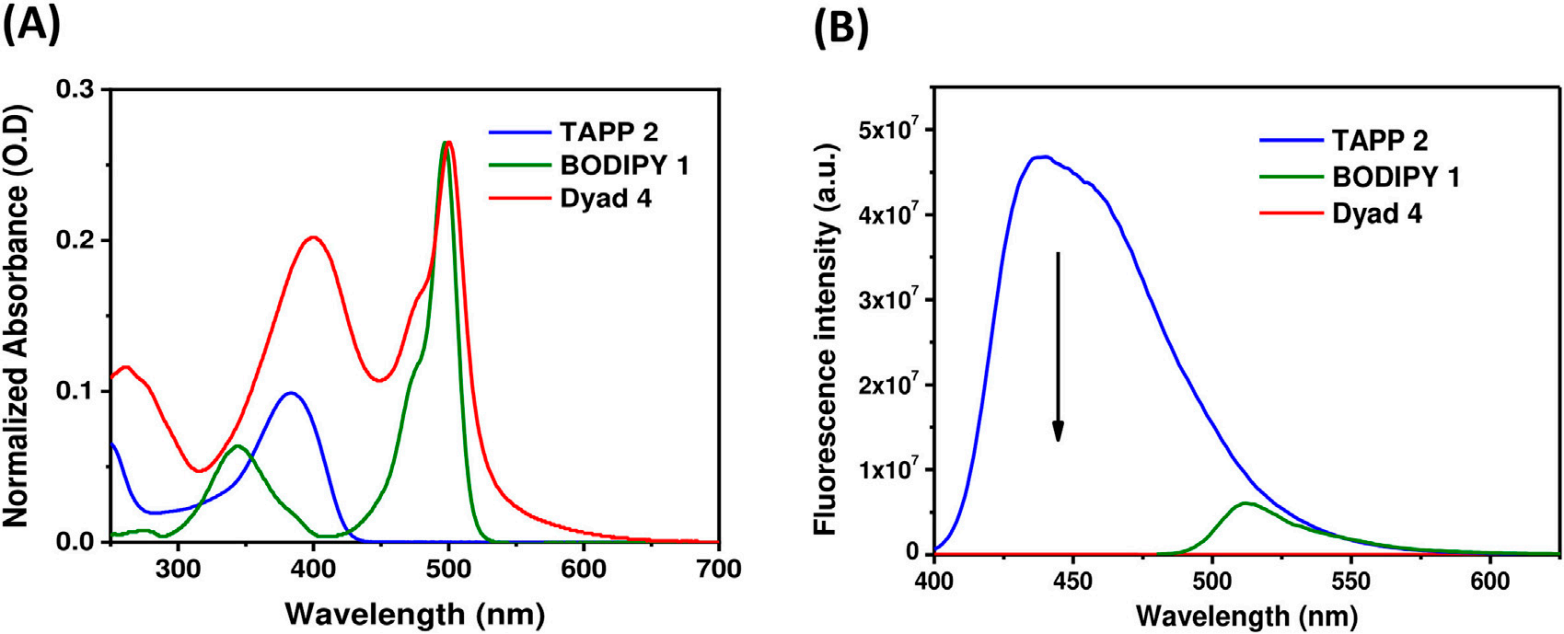
**(A)** UV-vis absorption spectra of TAPP **2**, BODIPY **1**, and dyad **4** in methanol (1.4–3.4 × 10^−6^ M); **(B)** Fluorescence spectra (O.D. corrected) of TAPP **2**, dyad **4** (*λ*_*ex*_ = 395 nm), and BODIPY **1** (*λ*_*ex*_ = 475 nm) in methanol.

**FIGURE 4:**
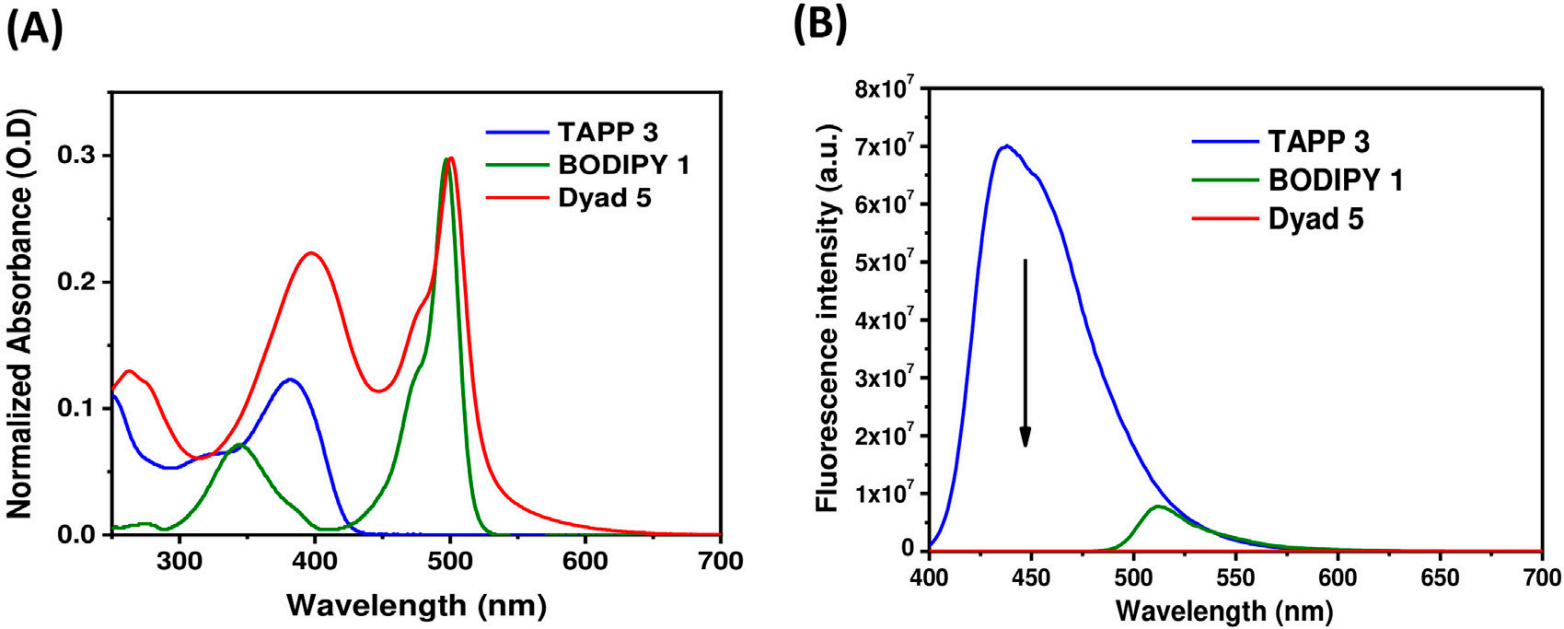
**(A)** UV-vis absorption spectra of TAPP **3**, BODIPY **1**, and dyad **5** in methanol (1.4–3.4 × 10^−6^ M); **(B)** Fluorescence spectra (O.D. corrected) of TAPP **3**, dyad **5** (*λ*_*ex*_ = 395 nm), and BODIPY **1** (*λ*_*ex*_ = 475 nm) in methanol.

Steady-state absorption and fluorescence properties of dyads **4** and **5** were recorded in methanol and compared with that of acceptor BODIPY **1** and donors TAPP **2** and **3**. Different photophysical parameters are tabulated in Table 2, and their absorption and fluorescence spectra are shown in Figures 3, 4, respectively.

**TABLE 2:** Absorption and emission of acceptor BODIPY **1**, donors TAPP **2**, **3**, and dyads **4**, **5** in methanol.

Compound	λ_abs_ (nm)	ε_max_ (M^−1^ cm^−1^)^a^	λ_em_ (nm)	ν (cm^−1^)^b^	Φ_fl_	% ETE
TAPP **2**	383	37,915	440	3,382	0.72^d^	-
TAPP **3**	381	36,169	439	3,468	0.71^d^	-
BODIPY **1**	497	35,471	516	741	0.0412^e^	-
Dyad **4**	400 500	53,105 69,840	516 516	5,620^c^ 620	0.0005^d^	99.83
Dyad **5**	397 500	72,887 94,723	517 533	5,847^c^ 1,238	0.0003^d^	99.79

^a^ Extinction co-efficient at λ_abs_.

^b^ Stokes shift.

^c^ Pseudo-Stokes shift.

^d^ Quantum yield of fluorescence measured using perylene in ethanol (Φ_fl_ = 0.92) as the reference (Crosby and Demas, 1971).

^e^ PM567 in ethanol (Φ_fl_ = 0.83) as the reference (Mula et al., 2008).

Donors TAPP **2** and **3** showed the longest absorption maximum (λ_abs_) at ∼382 nm and high blue fluorescence (Φ_fl_ = 0.72), with a fluorescence maximum (λ_fl_) at ∼440 nm; that is, the Stokes shifts of both the TAPPs are large (∼3,400 cm^−1^). On the other hand, the absorption and fluorescence spectra of acceptor BODIPY **1** are red-shifted compared to TAPP **2** and **3**. The λ_abs_ of BODIPY **1** is at 497 nm, and it showed greenish fluorescence with λ_em_ at 516 nm. Importantly, the fluorescence of BODIPY **1** is quenched (Φ_fl_ = 0.04) because of high non-radiative decay due to the free rotation of the C-8 phenyl ring (Sunahara et al., 2007).

Photophysical property studies of dyad **4** showed that absorption spectra contain λ_abs_ peaks of both donor and acceptor moieties at 400 nm and 500 nm, respectively (Figure 3; Table 2). This indicates no/less electronic conjugation between the TAPP (donor) and the BODIPY (acceptor) moieties as seen in the DFT optimized structure discussed *vide supra*. In fluorescence studies, when the TAPP moiety was excited at 395 nm, no characteristic blue fluorescence of the TAPP moiety or greenish-yellow fluorescence of BODIPY was observed. Complete quenching of TAPP fluorescence clearly indicates an efficient transfer of TAPP excitation energy to the acceptor BODIPY dyes. Due to the non-fluorescent nature of the acceptor BODIPY as discussed *vide supra*, emission of the BODIPY dyes was also not observed. The energy transfer efficiency was calculated from the decrease in the donor TAPP fluorescence, which showed extremely high energy transfer (99.83%) from TAPP to the BODIPY moiety.

Similar to dyad **4**, dyad **5** also showed absorption spectra containing λ_abs_ peaks of both donor and acceptor moieties at 397 nm and 500 nm, respectively (Figure 4; Table 2). These prove that in dyad **5**, the donor and acceptor moieties are not electronically conjugated as also predicted from DFT optimized structures discussed *vide supra*. While exciting dyad **5** at the donor part (395 nm), no fluorescence was observed either from the donor part or from the acceptor part. The drastic decrease in TAPP fluorescence clearly indicates an efficient transfer of TAPP excitation energy to the acceptor BODIPY dyes, as also seen in the case of dyad **4**. As the acceptor BODIPY is non-fluorescent in nature, emission of the BODIPY dyes was also not observed. The energy transfer efficiency was calculated from the decrease in the donor fluorescence, which showed extremely high energy transfer (99.79%) from TAPP to the BODIPY moiety.

### Viscosity sensing study of dyad 5

**FIGURE 5:**
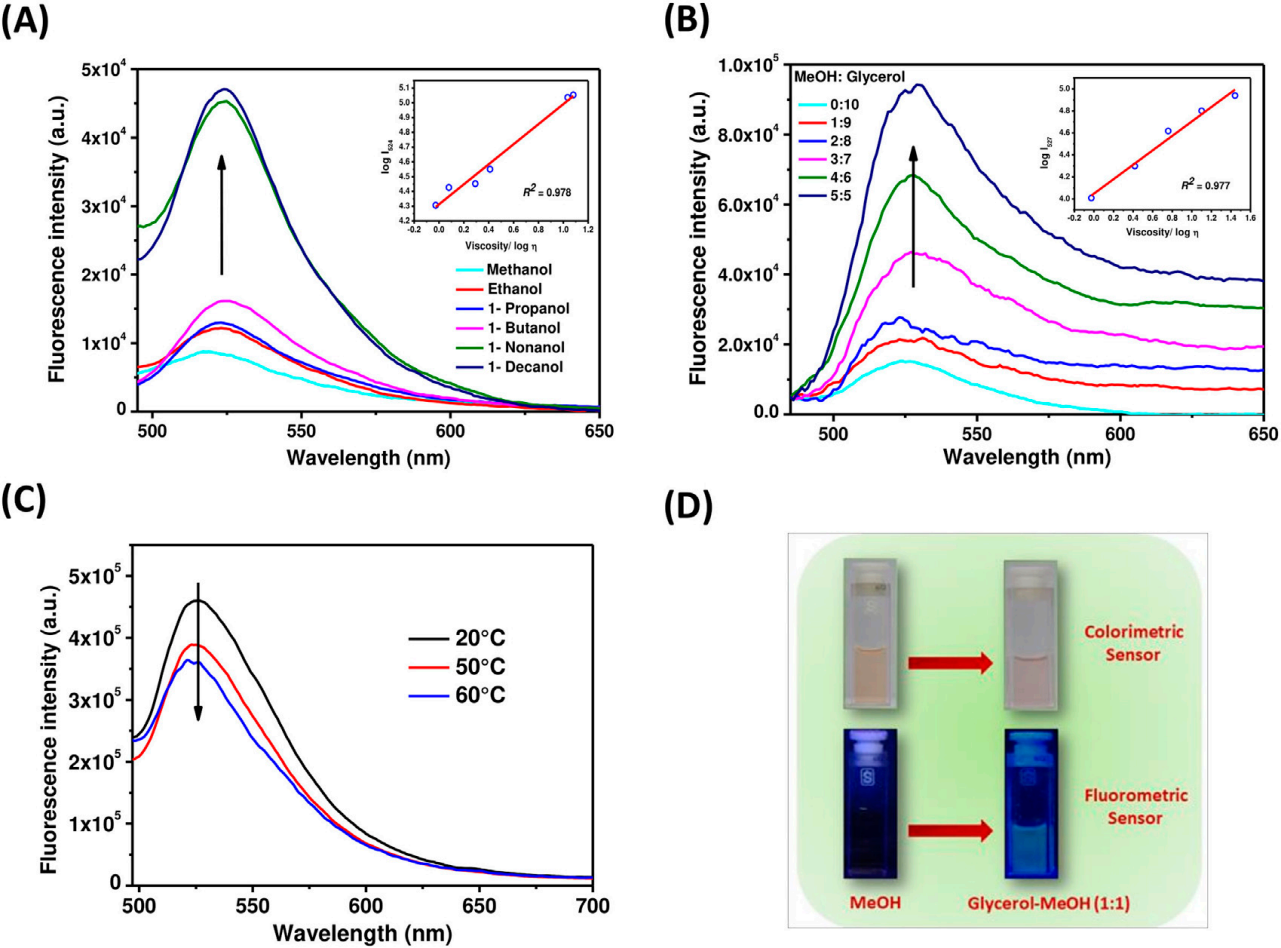
**(A)** Emission spectra of dyad **5** in methanol, ethanol, 1-propanol, 1-butanol, 1-nonanol, and 1-decanol (λ_ex_ = 395 nm) at 25°C; inset: The linear response between log I_524_ and log η in different n-alcohols. **(B)** Emission spectra of dyad **5** in different ratios of a glycerol-methanol mixture (λ_ex_ = 395 nm) at 25°C; inset: The linear response between log I_527_ and log η in different glycerol–methanol mixtures. **(C)** Temperature-dependent fluorescence of dyad **5** in a glycerol–methanol mixture (1:1) (λ_ex_ = 395 nm). **(D)** Color change of dyad **5** solutions in a methanol and glycerol–methanol mixture (1:1) under visible and UV light.

Next, the viscosity-sensing ability of these TAPP-BODIPY dyads was investigated. For this, the absorbance and fluorescence of dyad **5** were checked in solvents with higher viscosity, that is, in ethanol, 1-propanol, 1-butanol, 1-nonanol, 1-decanol, and methanol/glycerol mixtures, and these properties were compared with those determined in low polar methanol as discussed before. The absorption spectra of dyad **5** in different n-alcohols and glycerol-methanol (1:1) mixtures were similar to that of methanol (Supplementary Figure S1). However, remarkable changes were observed in fluorescence studies (Figures 5A, B). With excitation at the TAPP moiety (λ_ex_ = 395 nm), the fluorescence emission from the BODIPY moiety was increased sharply in both 1-decanol and the methanol/glycerol (1:1) mixture, showing greenish-yellow fluorescence (Figures 5A, B, D) compared to its non-fluorescent nature in methanol. In more viscous solvents, intramolecular rotation along C-C bonds connecting TAPP and BODIPY dyes decreases; thus, emission from the BODIPY moiety is enhanced. Importantly, fluorescence enhancement in both the solvents was similar when excited at TAPP as well as the BODIPY moiety in dyad **5**. This also confirmed the efficient energy transfer from the TAPP moiety to the BODIPY moiety. In different alcohols as well as in methanol/glycerol mixtures, the fluorescence intensities of dyad **5** increased with an increase in their viscosities. Interestingly, in both studies, the logarithm of fluorescent intensity and the logarithm of viscosity of solution obeyed a linear relationship as per the Förster–Hoffmann equation (Förster and Hoffmann, 1971; Koenig et al., 2016). Furthermore, the temperature-dependent fluorescence of dyad **5** was measured in methanol and a glycerol-methanol (1:1) mixture. It was observed that with increasing temperature of the glycerol-methanol (1:1) mixture, emission from the BODIPY moiety decreases (Figure 5C). (Sen et al., 2022) Now, with increasing temperature, the viscosity of the glycerol-methanol (1:1) mixture decreases; thus, the emission from the BODIPY moiety also decreases (Figure 5C). On the other hand, no/very little change in the fluorescence of dyad **5** is observed with increasing temperature in pure methanol, as the temperature-dependent viscosity change in methanol is negligible (Supplementary Figure S4). This clearly indicates that the fluorescence properties of dyad **5** depend on the viscosity of the medium. This showed the ability of dyad **5** to sense the viscosity as anticipated.

## Conclusion

Two TAPP-BODIPY dyads, **4** and **5**, were developed as molecular rotors in which two naked BODIPY dyes (acceptors) are linked with TAPP moieties (donors) through the C2 and C5 positions via phenylethynyl linkers. X-ray crystallographic and theoretical studies showed that both the TAPP and BODIPY moieties are twisted with respect to each other; that is, they are not electronically conjugated. This is also confirmed by spectroscopic studies. Fluorescence studies showed highly efficient energy transfer from the donor TAPP moiety to the acceptor BODIPY moiety on excitation at the TAPP part. Due to the non-fluorescent characteristics of the naked BODIPY dyes, no fluorescence emission was observed from the BODIPY moiety. With the increase in solvent viscosity, the free rotations of the BODIPY dyes were restricted, and high emissions from the BODIPY moieties were observed. For example, the absorbance and fluorescence of dyad **5** were checked in solvents with higher viscosity, that is, in n-alcohols and methanol/glycerol (1:1) systems. On excitation at the TAPP moiety (λ_ex_ = 395 nm), a remarkable greenish-yellow fluorescence was observed from the BODIPY moiety. Furthermore, with an increase in the temperature of the methanol/glycerol (1:1) system, the fluorescence started decreasing due to the lowering of the viscosity. All these observations confirmed that the dyad **5** is capable of sensing the viscosity of the medium via the FRET-based Off-On mechanism. This type of viscosity sensor with a very large pseudo-Stokes shift will be useful for advanced chemo-bio sensing and imaging applications.

## Experimental section

### General methods and materials

The detailed experimental methods and the data for the characterization of synthesized compounds (^1^H and ^13^C NMR spectrum) are given in the Supplementary Material.

### General procedure for the synthesis of TAPP 13 and 14

*p*-Methoxy/methyl aniline (1 mmol) and 4-((trimethylsilyl)ethynyl) benzaldehyde (1 mmol) were mixed in glacial acetic acid/toluene (1:10, 4 mL), and the mixture was heated at 50°C for 1 h in a 50 mL Schlenk tube. Then, Fe(ClO_4_)_3_·xH_2_O (0.03 mmol) and 2,3-butadione (0.5 mmol) were added, and the mixture was heated again at 90°C for 12 h. Next, the crude product was dried under reduced pressure and subjected to column chromatography (silica gel, DCM/petroleum ether, 30:70) to furnish the pure products TAPP **13/14,** respectively.

### 1,4-Bis(4-methoxyphenyl)-2,5-bis(4((trimethylsilyl)ethynyl)phenyl)-1,4-dihydropyrrolo[3,2-b]pyrrole (TAPP 13)

TAPP **13** was synthesized by following the general procedure using *p*-methoxyaniline (123 mg, 1.0 mmol), 4-((trimethylsilyl)ethynyl)benzaldehyde (202 mg, 1.0 mmol), 2,3-butadione (43 μL, 0.5 mmol), and Fe(ClO_4_)_3_ .H_2_O (11 mg, 0.03 mmol). Pure TAPP **13** was obtained as a yellow solid. Yield: 344 mg, (52%), R_f_ = 0.75 (DCM/hexane, 30:70, v/v); ^1^H NMR (500 MHz, CDCl_3_): δ 0.23 (s, 18H), 3.83 (s, 6H), 6.35 (s, 2H), 6.89 (d, *J* = 8.0 Hz, 4H), 7.12 (d, *J* = 7.0 Hz, 4H), 7.18 (d, *J* = 8.0 Hz, 4H), 7.30 (d, *J* = 8.5 Hz, 4H) ppm; ^13^C NMR (125 MHz, CDCl_3_): δ -0.03, 55.5, 94.1, 94.6, 105.3, 114.4, 120.2, 126.6, 127.5, 131.8, 132.5, 132.9, 133.6, 135.6, 157.7 ppm; HRMS (ESI/Q-TOF) m/z: [M + H]^+^ Calcd for C_42_H_43_N_2_O_2_Si_2_ 663.2857; Found 663.2821.

### 1,4-Bis(4-methylphenyl)-2,5-bis(4((trimethylsilyl)ethynyl)phenyl)-1,4-dihydropyrrolo[3,2-*b*]pyrrole (TAPP 14)

TAPP **14** was synthesized by following the general procedure using *p*-methylaniline (107 mg, 1.0 mmol), 4-((trimethylsilyl)ethynyl) benzaldehyde (202 mg, 1.0 mmol), 2,3-butanedione (43 μL, 0.5 mmol), and Fe(ClO_4_)_3_.H_2_O (11 mg, 0.03 mmol). Pure TAPP **14** was obtained as a yellow solid. Yield: 208 mg (66%); R_f_ = 0.45 (DCM/hexane, 30:70, v/v); ^1^H NMR (500 MHz, CDCl_3_): δ 0.23 (s, 18H), 2.37 (s, 6H), 6.38 (s, 2H), 7.12–7.15 (m, 12H), 7.30 (d, *J* = 8.1 Hz, 4H) ppm; ^13^C NMR (125 MHz, CDCl_3_): δ **-**0.03, 20.9, 94.5, 94.8, 105.3, 120.3, 125.1, 127.6, 129.8, 131.7, 132.4, 133.7, 135.5, 135.7, 137.3 ppm; HRMS (ESI/Q-TOF) m/z: [M + H]^+^ Calcd for C_42_H_43_N_2_Si_2_ 631.2959; Found 631.2964.

### General procedure for the synthesis of TAPP 2 and 3

TAPP **13/14** (0.16 mmol) and K_2_CO_3_ (0.5 mmol) were mixed in dry MeOH:DCM (1:1, 10 mL), and the mixture was stirred at room temperature for 24 h. The reaction mixture turned yellow, was extracted with dichloromethane (3 × 30 mL), and washed with saturated aq. NaHCO_3_ (30 mL), brine (30 mL) and water (30 mL). The organic layer was dried over anhydrous Na_2_SO_4_ and evaporated under reduced pressure. The crude product was purified by column chromatography (silica gel, DCM/petroleum ether, 50:50) to furnish the pure products TAPP **2/3**, respectively.

### 2,5-Bis(4-ethynylphenyl)-1,4-bis(4-methoxyphenyl)-1,4-dihydropyrrolo[3,2-*b*]pyrrole (TAPP 2)

TAPP **2** was synthesized by following the general procedure using TAPP **13** (105 mg, 0.16 mmol) and K_2_CO_3_ (68 mg, 0.5 mmol). Pure TAPP **2** was obtained as a yellow solid. Yield: 67 mg (80%); R_f_ = 0.64 (DCM/hexane, 40:60, v/v); ^1^H NMR (500 MHz, CDCl_3_): δ 3.08 (s, 2H), 3.84 (s, 6H), 6.36 (s, 2H), 6.91 (d, *J* = 9.0 Hz, 4H), 7.15 (d, *J* = 8.5 Hz, 4H), 7.20 (d, *J* = 9.0 Hz, 4H), 7.33 (d, *J* = 8.5 Hz, 4H) ppm; ^13^C NMR (125 MHz, CDCl_3_): δ 55.5, 77.5, 83.8, 94.3, 114.5, 119.3, 126.6, 127.6, 131.9, 132.6, 132.9, 134.0, 135.6, 157.8 ppm; HRMS (ESI/Q-TOF) m/z: [M + H]^+^ Calcd for C_36_H_27_N_2_O_2_ 519.2067; Found 519.2076.

### 2,5-Bis(4-ethynylphenyl)-1,4-di-(4-methylphenyl)-1,4-dihydropyrrolo[3,2-*b*]pyrrole (TAPP 3)

TAPP **3** was synthesized by following the general procedure using TAPP **14** (101 mg, 0.16 mmol) and K_2_CO_3_ (69 mg, 0.5 mmol). Pure TAPP **3** was obtained as a yellow solid. Yield: 73 mg (94%); R_f_ = 0.46 (DCM/hexane, 30:70, v/v); ^1^H NMR (500 MHz, CDCl_3_): δ 2.38 (s, 6H), 3.07 (s, 2H)s, 6.39 (s, 2H), 7.15–7.17 (m, 12H), 7.33 (d, *J* = 8.3 Hz, 4H) ppm; ^13^C NMR (125 MHz, CDCl_3_): δ 21.0, 77.5, 83.8, 94.9, 119.3, 125.1, 127.6, 129.8, 131.9, 132.4, 134.0, 135.4, 135.8, 137.3 ppm; HRMS (ESI/Q-TOF) m/z: [M + H]^+^ Calcd for C_36_H_27_N_2_ 487.2168; Found 487.2163.

### 4,4-Difluoro-8-(4′-iodophenyl)-4-bora-3a,4a-diaza-s-indecene (9)

A mixture of 4-iodobenzaldehyde (**7**) (700 mg, 3.0 mmol), an excess of pyrrole (**6**) (9 mL, 120.6 mmol), and trifluoroacetic acid (5 drops) was stirred at 25°C for 1 day. Excess pyrrole was distilled off, and the residue was purified by flash column chromatography (silica gel, ethyl acetate/petroleum ether, 20:80) to obtain dipyrromethane **8** (770 mg, 73%) as a cream color solid. Then, **8** (770 mg, 2.2 mmol) was dissolved in dry DCM, DDQ (753 mg, 3.3 mmol) was added into it, and the resulting mixture was stirred at 25°C for 4 h. Next, NEt_3_ (1.8 mL, 13 mmol) and BF_3_.OEt_2_ (1.6 mL, 13 mmol) were added, and stirring was continued for another 12 h. The reaction mixture was quenched with sat. NaHCO_3_ solution (50 mL), extracted with dichloromethane (100 mL), washed with water (3 × 25 mL), and dried with Na_2_SO_4_. The organic layer was concentrated *in vacuo*, and the crude product was purified by flash column chromatography (silica gel, ethyl acetate/petroleum ether, 5:95) to obtain BODIPY **9** as a dark orange solid (Betancourt-Mendiola et al., 2015). Yield: 159 mg (18%); R_f_ = 0.65 (ethyl acetate/hexane, 25: 75, v/v); ^1^H NMR (500 MHz, CDCl_3_): δ 6.56 (d, *J* = 4.2 Hz, 2H), 6.90 (d, *J* = 4.3 Hz, 2H), 7.30 (d, *J* = 8.3 Hz, 2H), 7.89 (d, *J* = 8.3 Hz, 2H), 7.95 (s, 2H) ppm; ^13^C NMR (125 MHz, CDCl_3_): δ 97.5, 118.8, 131.3, 131.9, 133.1, 134.6, 137.7, 144.5, 145.9 ppm; HRMS (ESI/Q-TOF) m/z: [M + H]^+^ Calcd for C_15_H_11_BF_2_IN_2_ 395.0022; Found 395.0021.

### General procedure for the synthesis of dyads 4 and 5

TAPP **2**/**3** (0.05 mmol), BODIPY **9** (0.10 mmol), and di-isopropyl amine (0.5 mL) were dissolved in dry THF (6 mL), and the solution was degassed properly. Pd(PPh_3_)_2_Cl_2_ (3.5 mg, 0.005 mmol) and CuI (1 mg, 0.005 mmol) were added, and the reaction was stirred at 25°C for 24 h. Removal of the solvent *in vacuo* followed by column chromatography of the residue (silica gel, DCM/petroleum ether, 70:30) furnished dyads **4**/**5**, respectively.

### Dyad 4

Dyad **4** was synthesized by following the general procedure using TAPP **2** (26 mg, 0.05 mmol) and BODIPY **1** (42 mg, 0.1 mmol). Pure dyad **4** was obtained as a dark red solid. Yield: 40 mg (80%). R_f_ = 0.35 (DCM/hexane, 70:30, v/v); ^1^H NMR (500 MHz, CDCl_3_): δ 3.86 (s, 6H), 6.42 (s, 2H), 6.57 (d, *J* = 7.4 Hz, 4H), 6.93–6.96 (m, 8H), 7.23–7.24 (m, 8H), 7.42 (d, *J* = 7.4 Hz, 4H), 7.56 (d, *J* = 7.7 Hz, 4H), 7.65 (d, *J* = 7.7 Hz, 4H), 7.96 (s, 4H) ppm; ^13^C NMR (125 MHz, CDCl_3_): δ 55.5, 88.9, 92.6, 94.4, 114.5, 118.7, 119.8, 126.3, 126.7, 127.7, 130.6, 131.4, 131.5, 131.6, 132.9, 133.3, 134.7, 144.3, 146.5, 157.9 ppm; MS (MALDI-TOF): m/z [M]^+^ Calcd for C_66_H_44_B_2_F_4_N_6_O_2_: 1,050.4; Found 1,050.1.

### Dyad 5

Dyad **5** was synthesized by following the general procedure using TAPP **3** (25 mg, 0.05 mmol) and BODIPY **1** (42 mg, 0.10 mmol). Pure dyad **5** was obtained as a dark red solid. Yield: 24 mg (48%); R_f_ = 0.45 (DCM/hexane, 70:30, v/v); ^1^H NMR (500 MHz, CDCl_3_): δ 2.41 (s, 6H), 6.45 (s, 2H), 6.56 (d, *J* = 7.4 Hz, 4H), 6.95 (d, *J* = 7.6 Hz, 4H), 7.19–7.21 (m, 8H), 7.23 (d, *J* = 8.1 Hz, 4H), 7.42 (d, *J* = 8.1 Hz, 4H), 7.56 (d, *J* = 7.9 Hz, 4H), 7.65 (d, *J* = 7.9 Hz, 4H), 7.95 (s, 4H) ppm; ^13^C NMR (125 MHz, CDCl_3_): δ 21.0, 88.9, 92.6, 94.9, 118.7, 119.8, 125.1, 126.3, 127.7, 129.9, 130.5, 131.3, 131.4, 131.5, 132.6, 133.3, 133.9, 134.7, 135.5, 135.8, 137.3, 144.2, 146.4 ppm. HRMS (ESI/Q-TOF) m/z: [M + H]^+^ Calcd for C_66_H_45_B_2_F_4_N_6_ 1,019.3822; Found 1,019.3820.

## Data Availability

The original contributions presented in the study are included in the article/Supplementary Material; further inquiries can be directed to the corresponding author.
