# Supplementary material for: Tetraarylpyrrolo[3,2-b]pyrrole-BODIPY dyad: a molecular rotor for FRET-based viscosity sensing
Source: Front Chem. 2024 Oct 10;12:1473769. doi: 10.3389/fchem.2024.1473769 (PMC11499138; doi:10.3389/fchem.2024.1473769)
Supplement: Supplementary file 1 [file DataSheet1.doc]

**Tetraarylpyrrolo[3,2-*b*]pyrrole-BODIPY dyad:**

**A Molecular Rotor for FRET based Viscosity Sensing**

Richa Agrawal^a,b^, Sudip Gorai^a,b^, Sunil Suresh Yadav^c^, Amey P. Wadawale^d^ and

Soumyaditya Mula^a,b^*

^a^Bio-Organic Division, Bhabha Atomic Research Centre, Mumbai-400085, India.

^b^Homi Bhabha National Institute, Anushakti Nagar, Mumbai-400094, India.

^C^National Centre for Nanoscience and Nanotechnology, University of Mumbai,

Mumbai-400098, India

^d^Chemistry Division, Bhabha Atomic Research Centre, Mumbai-400085, India.

Email: smula@barc.gov.in

**Table of Contents**

| **S. No.** | **Title** | **Page** |
| --- | --- | --- |
| 1 | General methods and materials | S3 |
| 2 | Photophysical Studies | S4-S7 |
| 3 | Crystallographic studies of TAPP 2 and 3 | S8-S21 |
| 4 | DFT Calculations | S22 |
| 5 | ^1^H NMR & ^13^C NMR Spectra | S23- S36 |
| 6 | References | S37 |

1. **General methods and materials**

All chemical reactions were carried out under argon atmosphere using anhydrous solvents in screw-cap Schlenk tube or round bottom flask with rubber septa. The chemicals used were obtained from commercial suppliers, only triethylamine (TEA) used after purification. The solvents like TEA, DCM, DCE were dried by distillation process over calcium hydride and THF was purified over sodium-benzophenone under argon atmosphere. All other solvents like ethanol, methanol, petroleum ether, chloroform and ethyl acetate used were from reputed companies of Analytical grade and used without purification. Reactions were monitored by thin-layer chromatography which was performed in commercially available 0.25 mm fluorescent silica gel plate (F-254) and visualized using a UV lamp (254 nm wavelength) or developed in iodine chamber or in alkaline KMnO_4_ solution after heating.

SC-XRD data was obtained using CrysAlisPro 1.171.40.57a (Rigaku Oxford Diffraction, 2019). ^1^H &^13^C NMR were recorded in 500 MHz Varian FT-NMR instruments. High Resolution Mass Spectrometric (HRMS) analysis was performed using either 6540 UHD Accurate-Mass Agilent Q-TOF LC/MS instrument or Bruker Maxis Impact Q-TOF LC/MS instrument. MALDI-TOF Mass Spectra was recorded in Bruker Auto flex Max TOF instrument. Spectroscopic data was taken from JASCO V-670 spectrophotometer & JASCO FP-6500 spectrofluorometer.

1. **Photophysical studies**

**Figure S1.** Absorption spectra of the dyad **5** in n-alcohols (3.2 X 10^-6^ M) at 25 °C**.**

**Figure S2.** Emission spectra of the dyad **5** in methanol and 1-decanol at λ_ex_ = 490 nm at 25 °C**.**

**Figure S3.** Emission spectra of the dyad **5** in methanol and glycerol-methanol (1:1) mixture at λ_ex_ = 490 nm at 25 °C**.**

**Figure S4.** Temperature dependent fluorescence of dyad **5** in methanol at λ_ex_ = 395 nm.

1. **Crystallographic studies of TAPP 2 and 3**

The crystallographic data for TAPP **2** and **3** were collected from a single crystal at 298(2) K using Cu Kα radiation (λ = 1.54184 Å) on a XtaLAB Synergy, Dualflex, HyPix four-circle diffractometer with a micro-focus sealed X-ray tube using mirror as monochromator and a HyPix detector. All data were integrated and a multi-scan absorption correction was applied using CrysAlis PRO.^[1]^ The structure were solved by dual methods using SHELXT and refined by full-matrix least-squares methods against *F*^2^ by olex2.refine.^[2,3]^ All non-hydrogen atoms were refined with anisotropic displacement parameters. The hydrogen atoms were refined isotropically on calculated positions using a riding model with their *U*_iso_ values constrained to 1.5 times the *U*_eq_ of their pivot atoms for terminal sp^3^ carbon atoms and 1.2 times for all other carbon atoms. Crystallographic data (including structure factors) for the structures reported in this paper have been deposited with the Cambridge Crystallographic Data Centre. ORTEP was employed for the final data presentation and structure plots.^[4]^ CCDC 2374116 and 2374117 contains the supplementary crystallographic data for this paper.

## Table S1. Crystal data and structure refinement for TAPP 2 and 3.

|  | TAPP 2 | **TAPP 3** |
| --- | --- | --- |
| CCDC number | 2374117 | 2374116 |
| Empirical formula | C_42_H_38_N_2_O_2_ | C_36_H_26_N_2_ |
| Formula weight | 602.782 | 486.621 |
| Temperature [K] | 298.15 | 298 |
| Crystal system | triclinic | triclinic |
| Space group (number) | 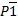(2) | 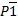(2) |
| *a* [Å] | 5.9575(1) | 5.9026(2) |
| *b* [Å] | 11.2692(2) | 10.8363(3) |
| *c* [Å] | 12.9233(2) | 11.3173(3) |
| α [Å] | 87.199(1) | 75.576(2) |
| β [Å] | 78.430(1) | 85.296(2) |
| γ [Å] | 81.581(2) | 78.402(3) |
| Volume [Å^3^] | 840.66(3) | 686.36(4) |
| *Z* | 1 | 1 |
| *ρ*_calc_ [g/cm^3^] | 1.191 | 1.177 |
| *μ* [mm^-1^] | 0.566 | 0.524 |
| *F*(000) | 320.976 | 256.756 |
| Crystal size [mm^3^] | 0.15×0.04×0.02 | 0.1×0.015×0.01 |
| Crystal colour | yellow | yellow |
| Crystal shape | needle | plate |
| Radiation | Cu *K_α_* (λ=1.54184 Å) | Cu *K_α_* (λ=1.54184 Å) |
| 2ϴ range [°] | 6.98 to 154.04 (0.79 Å) | 8.08 to 154.26 (0.79 Å) |
| Index ranges | -7 ≤ h ≤ 7 -14 ≤ k ≤ 14 -16 ≤ l ≤ 16 | -7 ≤ h ≤ 7 -13 ≤ k ≤ 13 -13 ≤ l ≤ 13 |
| Reflections collected | 20088 | 7400 |
| Independent reflections | 3421 *R*_int_ = 0.0618 *R*_sigma_ = 0.0319 | 2723 *R*_int_ = 0.0381 *R*_sigma_ = 0.0489 |
| Completeness to  θ = 67.6840° | 99.8 % | 99.0 % |
| Data / Restraints / Parameters | 3421/0/237 | 2723/0/173 |
| Goodness-of-fit on *F*^2^ | 1.2033 | 1.0312 |
| Final *R* indexes  [*I*≥2σ(*I*)] | *R*_1_ = 0.0541 w*R*_2_ = 0.1991 | *R*_1_ = 0.0492 w*R*_2_ = 0.1300 |
| Final *R* indexes  [all data] | *R*_1_ = 0.1036 w*R*_2_ = 0.2835 | *R*_1_ = 0.0593 w*R*_2_ = 0.1403 |
| Largest peak/hole [eÅ^3^] | 0.33/-0.40 | 0.17/-0.23 |

## Table S2. Bond lengths and angles for shelx of TAPP 2.

| **TAPP 2**  **Atom–Atom** | **Length [Å]** |
| --- | --- |
| N1–C2 | 1.399(2) |
| N1–C3 | 1.383(2) |
| N1–C12 | 1.427(2) |
| O1–C15 | 1.358(2) |
| O1–C18 | 1.426(4) |
| C1–H1 | 0.9300 |
| C1–C2 | 1.382(2) |
| C1–C3^#1^ | 1.408(2) |
| C2–C4 | 1.469(2) |
| C3–C3^#1^ | 1.371(3) |
| C4–C5 | 1.402(3) |
| C4–C9 | 1.388(3) |
| C5–H5 | 0.9300 |
| C5–C6 | 1.381(3) |
| C6–H6 | 0.9300 |
| C6–C7 | 1.389(3) |
| C7–C8 | 1.389(3) |
| C7–C10 | 1.443(3) |
| C8–H8 | 0.9300 |
| C8–C9 | 1.385(3) |
| C9–H9 | 0.9300 |
| C10–C11 | 1.160(3) |
| C11–H11 | 0.9300 |
| C12–C13 | 1.375(3) |
| C12–C17 | 1.381(3) |
| C13–H13 | 0.9300 |
| C13–C14 | 1.383(3) |
| C14–H14 | 0.9300 |
| C14–C15 | 1.380(3) |
| C15–C16 | 1.390(3) |
| C16–H16 | 0.9300 |
| C16–C17 | 1.375(3) |
| C17–H17 | 0.9300 |
| C18–H18a | 0.9600 |
| C18–H18b | 0.9600 |
| C18–H18c | 0.9600 |
| C19–H19a | 0.9700 |
| C19–H19b | 0.9700 |
| C19–C20 | 1.465(15) |
| C19–C21^#2^ | 1.541(11) |
| C20–H20a | 0.9700 |
| C20–H20b | 0.9700 |
| C20–C21 | 1.472(10) |
| C21–H21a | 0.9700 |
| C21–H21b | 0.9700 |
| C19A–H19c | 0.9700 |
| C19A–H19d | 0.9700 |
| C19A–C20A | 1.52(2) |
| C19A–C21A^#2^ | 1.541(18) |
| C20A–H20c | 0.9700 |
| C20A–H20d | 0.9700 |
| C20A–C21A | 1.466(16) |
| C21A–H21c | 0.9700 |
| C21A–H21d | 0.9700 |
| **Atom–Atom–Atom** | **Angle [°]** |
| C3–N1–C2 | 107.32(14) |
| C12–N1–C2 | 128.97(14) |
| C12–N1–C3 | 122.17(14) |
| C18–O1–C15 | 118.0(2) |
| C2–C1–H1 | 127.06(10) |
| C3^#1^–C1–H1 | 127.06(10) |
| C3^#1^–C1–C2 | 105.89(15) |
| C1–C2–N1 | 109.30(15) |
| C4–C2–N1 | 123.79(15) |
| C4–C2–C1 | 126.64(16) |
| C1^#1^–C3–N1 | 142.51(16) |
| C3^#1^–C3–N1 | 108.11(19) |
| C3^#1^–C3–C1^#1^ | 109.38(18) |
| C5–C4–C2 | 118.60(16) |
| C9–C4–C2 | 123.22(16) |
| C9–C4–C5 | 118.08(16) |
| H5–C5–C4 | 119.67(11) |
| C6–C5–C4 | 120.65(17) |
| C6–C5–H5 | 119.67(11) |
| H6–C6–C5 | 119.60(11) |
| C7–C6–C5 | 120.81(17) |
| C7–C6–H6 | 119.60(11) |
| C8–C7–C6 | 118.85(17) |
| C10–C7–C6 | 120.88(18) |
| C10–C7–C8 | 120.27(19) |
| H8–C8–C7 | 119.83(12) |
| C9–C8–C7 | 120.35(19) |
| C9–C8–H8 | 119.83(12) |
| C8–C9–C4 | 121.25(18) |
| H9–C9–C4 | 119.37(11) |
| H9–C9–C8 | 119.37(12) |
| C11–C10–C7 | 179.7(3) |
| H11–C11–C10 | 180.0 |
| C13–C12–N1 | 120.61(16) |
| C17–C12–N1 | 120.25(16) |
| C17–C12–C13 | 119.05(17) |
| H13–C13–C12 | 119.50(11) |
| C14–C13–C12 | 120.99(18) |
| C14–C13–H13 | 119.50(12) |
| H14–C14–C13 | 120.02(12) |
| C15–C14–C13 | 119.95(19) |
| C15–C14–H14 | 120.02(12) |
| C14–C15–O1 | 125.1(2) |
| C16–C15–O1 | 115.83(19) |
| C16–C15–C14 | 119.02(18) |
| H16–C16–C15 | 119.77(11) |
| C17–C16–C15 | 120.47(18) |
| C17–C16–H16 | 119.77(12) |
| C16–C17–C12 | 120.46(18) |
| H17–C17–C12 | 119.77(11) |
| H17–C17–C16 | 119.77(12) |
| H18a–C18–O1 | 109.5 |
| H18b–C18–O1 | 109.5 |
| H18b–C18–H18a | 109.5 |
| H18c–C18–O1 | 109.5 |
| H18c–C18–H18a | 109.5 |
| H18c–C18–H18b | 109.5 |
| H19b–C19–H19a | 107.9 |
| C20–C19–H19a | 109.2(6) |
| C20–C19–H19b | 109.2(5) |
| C21^#2^–C19–H19a | 109.2(6) |
| C21^#2^–C19–H19b | 109.2(4) |
| C21^#2^–C19–C20 | 112.3(7) |
| H20a–C20–C19 | 109.4(6) |
| H20b–C20–C19 | 109.4(5) |
| H20b–C20–H20a | 108.0 |
| C21–C20–C19 | 111.1(6) |
| C21–C20–H20a | 109.4(5) |
| C21–C20–H20b | 109.4(4) |
| C20–C21–C19^#2^ | 114.9(6) |
| H21a–C21–C19^#2^ | 108.6(6) |
| H21a–C21–C20 | 108.6(5) |
| H21b–C21–C19^#2^ | 108.6(7) |
| H21b–C21–C20 | 108.6(5) |
| H21b–C21–H21a | 107.5 |
| H19d–C19A–H19c | 108.1 |
| C20A–C19A–H19c | 109.6(9) |
| C20A–C19A–H19d | 109.6(8) |
| C21A^#2^–C19A–H19c | 109.6(8) |
| C21A^#2^–C19A–H19d | 109.6(9) |
| C21A^#2^–C19A–C20A | 110.3(13) |
| H20c–C20A–C19A | 109.4(9) |
| H20d–C20A–C19A | 109.4(10) |
| H20d–C20A–H20c | 108.0 |
| C21A–C20A–C19A | 111.1(10) |
| C21A–C20A–H20c | 109.4(7) |
| C21A–C20A–H20d | 109.4(7) |
| C20A–C21A–C19A^#2^ | 113.3(13) |
| H21c–C21A–C19A^#2^ | 108.9(8) |
| H21c–C21A–C20A | 108.9(7) |
| H21d–C21A–C19A^#2^ | 108.9(10) |
| H21d–C21A–C20A | 108.9(6) |
| H21d–C21A–H21c | 107.7 |

Symmetry transformations used to generate equivalent atoms:
#1: 1-X, 1-Y, 1-Z; #2: 1-X, 2-Y, 1-Z.

## Table S3. Bond lengths and angles for shelx of TAPP 3.

| **TAPP 3**  **Atom-Atom** | **Length [Å]** |
| --- | --- |
| N1–C12 | 1.4318(16) |
| N1–C11 | 1.3824(16) |
| N1–C9 | 1.4019(18) |
| C12–C17 | 1.3794(19) |
| C12–C13 | 1.375(2) |
| C11–C11^#1^ | 1.376(3) |
| C11–C10^#1^ | 1.403(2) |
| C6–C9 | 1.4657(18) |
| C6–C7 | 1.400(2) |
| C6–C5 | 1.400(2) |
| C9–C10 | 1.3866(18) |
| C10–H10 | 0.9300 |
| C7–H7 | 0.9300 |
| C7–C8 | 1.374(2) |
| C17–H17 | 0.9300 |
| C17–C16 | 1.382(2) |
| C3–C8 | 1.392(2) |
| C3–C4 | 1.390(2) |
| C3–C2 | 1.438(2) |
| C8–H8 | 0.9300 |
| C5–H5 | 0.9300 |
| C5–C4 | 1.378(2) |
| C13–H13 | 0.9300 |
| C13–C14 | 1.382(2) |
| C4–H4 | 0.9300 |
| C15–C16 | 1.383(2) |
| C15–C14 | 1.381(2) |
| C15–C18 | 1.510(2) |
| C16–H16 | 0.9300 |
| C14–H14 | 0.9300 |
| C2–C1 | 1.168(2) |
| C1–H1 | 0.9300 |
| C18–H18a | 0.9600 |
| C18–H18b | 0.9600 |
| C18–H18c | 0.9600 |
| **Atom–Atom-Atom** | **Angle [°]** |
| C11–N1–C12 | 123.51(11) |
| C9–N1–C12 | 127.95(11) |
| C9–N1–C11 | 107.59(10) |
| C17–C12–N1 | 120.69(12) |
| C13–C12–N1 | 119.53(12) |
| C13–C12–C17 | 119.77(12) |
| C11^#1^–C11–N1 | 107.78(15) |
| C10^#1^–C11–N1 | 142.54(12) |
| C7–C6–C9 | 120.11(12) |
| C5–C6–C9 | 122.60(13) |
| C5–C6–C7 | 117.25(13) |
| C6–C9–N1 | 123.11(11) |
| C10–C9–N1 | 109.06(12) |
| C10–C9–C6 | 127.58(13) |
| C9–C10–C11^#1^ | 105.90(12) |
| H10–C10–C11^#1^ | 127.05(7) |
| H10–C10–C9 | 127.05(8) |
| H7–C7–C6 | 119.27(8) |
| C8–C7–C6 | 121.46(13) |
| C8–C7–H7 | 119.27(9) |
| H17–C17–C12 | 120.24(8) |
| C16–C17–C12 | 119.53(14) |
| C16–C17–H17 | 120.24(9) |
| C4–C3–C8 | 118.10(13) |
| C2–C3–C8 | 121.88(14) |
| C2–C3–C4 | 120.02(13) |
| C3–C8–C7 | 120.90(14) |
| H8–C8–C7 | 119.55(9) |
| H8–C8–C3 | 119.55(9) |
| H5–C5–C6 | 119.48(9) |
| C4–C5–C6 | 121.04(14) |
| C4–C5–H5 | 119.48(9) |
| H13–C13–C12 | 120.00(8) |
| C14–C13–C12 | 120.00(14) |
| C14–C13–H13 | 120.00(9) |
| C5–C4–C3 | 121.19(13) |
| H4–C4–C3 | 119.40(8) |
| H4–C4–C5 | 119.40(9) |
| C14–C15–C16 | 117.77(13) |
| C18–C15–C16 | 121.25(16) |
| C18–C15–C14 | 120.98(17) |
| C15–C16–C17 | 121.63(14) |
| H16–C16–C17 | 119.19(9) |
| H16–C16–C15 | 119.19(8) |
| C15–C14–C13 | 121.29(15) |
| H14–C14–C13 | 119.36(9) |
| H14–C14–C15 | 119.36(9) |
| C1–C2–C3 | 178.55(18) |
| H1–C1–C2 | 180.0 |
| H18a–C18–C15 | 109.5 |
| H18b–C18–C15 | 109.5 |
| H18b–C18–H18a | 109.5 |
| H18c–C18–C15 | 109.5 |
| H18c–C18–H18a | 109.5 |
| H18c–C18–H18b | 109.5 |
| H4–C4–C3 | 119.40(8) |
| H4–C4–C5 | 119.40(9) |
| C14–C15–C16 | 117.77(13) |
| C18–C15–C16 | 121.25(16) |
| C18–C15–C14 | 120.98(17) |
| C15–C16–C17 | 121.63(14) |
| H16–C16–C17 | 119.19(9) |
| H16–C16–C15 | 119.19(8) |
| C15–C14–C13 | 121.29(15) |
| H14–C14–C13 | 119.36(9) |
| H14–C14–C15 | 119.36(9) |
| C1–C2–C3 | 178.55(18) |
| H1–C1–C2 | 180.0 |
| H18a–C18–C15 | 109.5 |
| H18b–C18–C15 | 109.5 |
| H18b–C18–H18a | 109.5 |
| H18c–C18–C15 | 109.5 |
| H18c–C18–H18a | 109.5 |
| H18c–C18–H18b | 109.5 |

Symmetry transformations used to generate equivalent atoms:
#1: 2-X, 1-Y, -Z.

1. **DFT calculations**

**Table S4.** HOMO and LUMO surfaces of dyad **4** and dyad **5.**

| **Dyads** | **Optimised Structures** | **LUMO+1** | **LUMO** |
| --- | --- | --- | --- |
| **4** | **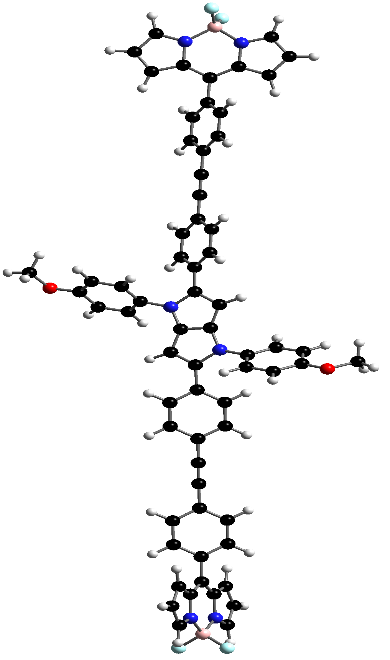** | 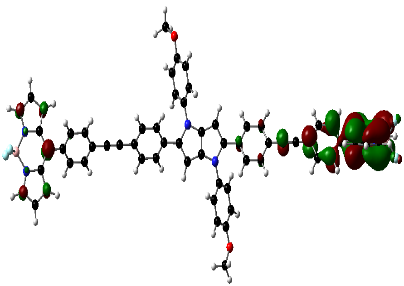~~-~~3.01339eV | 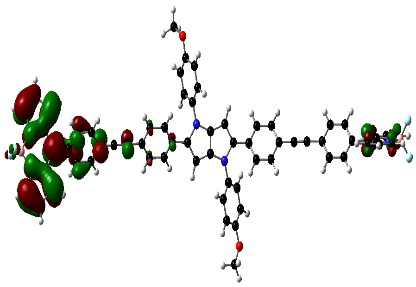-3.04523 eV |
|  |  | **HOMO** | **HOMO-1** |
|  |  | 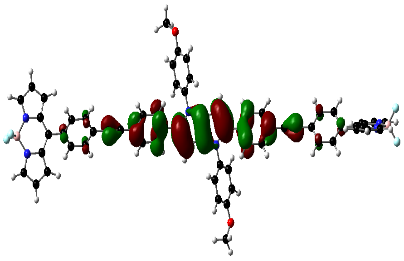-4.98132eV | 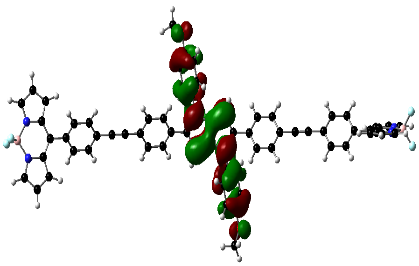-5.59711 eV |
|  |  | **LUMO+1** | **LUMO** |
| **5** | 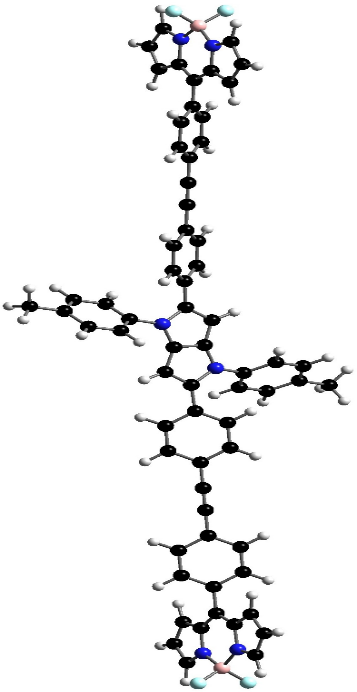 | 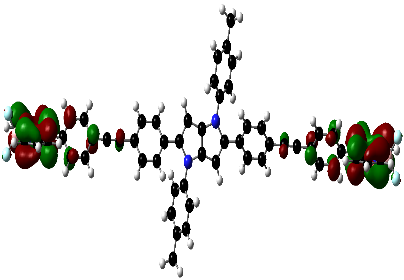-3.02591 eV | 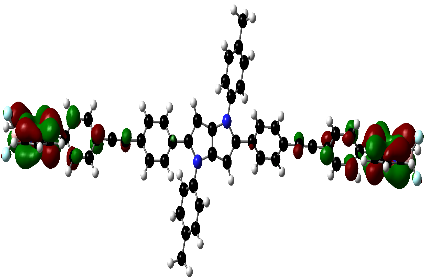-3.04169eV |
|  |  | **HOMO** | **HOMO-1** |
|  |  | 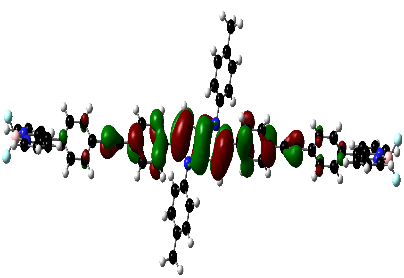-5.00907 eV | 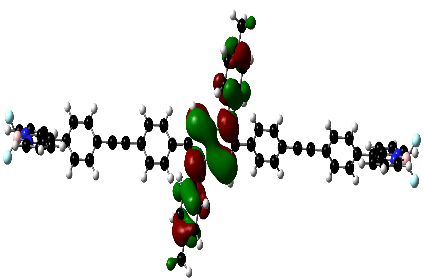-5.70324eV |

1. **^1^H NMR & ^13^C NMR Spectra**

**
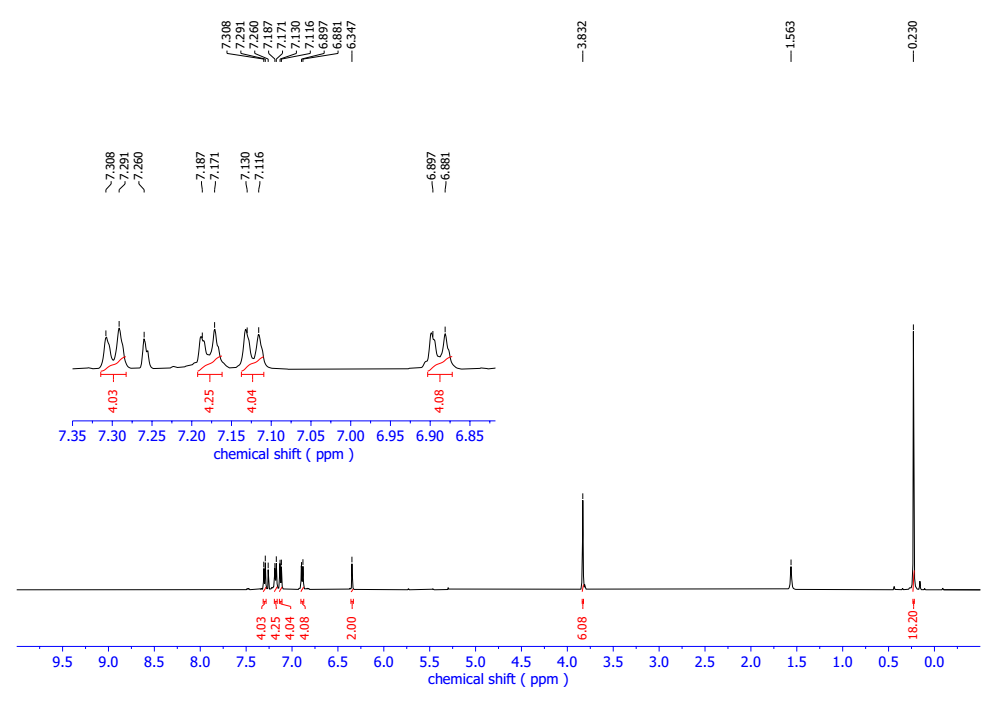
**

**Figure S5**. ^1^H NMR (500 MHz, CDCl_3_) Spectrum of TAPP **13.**

**
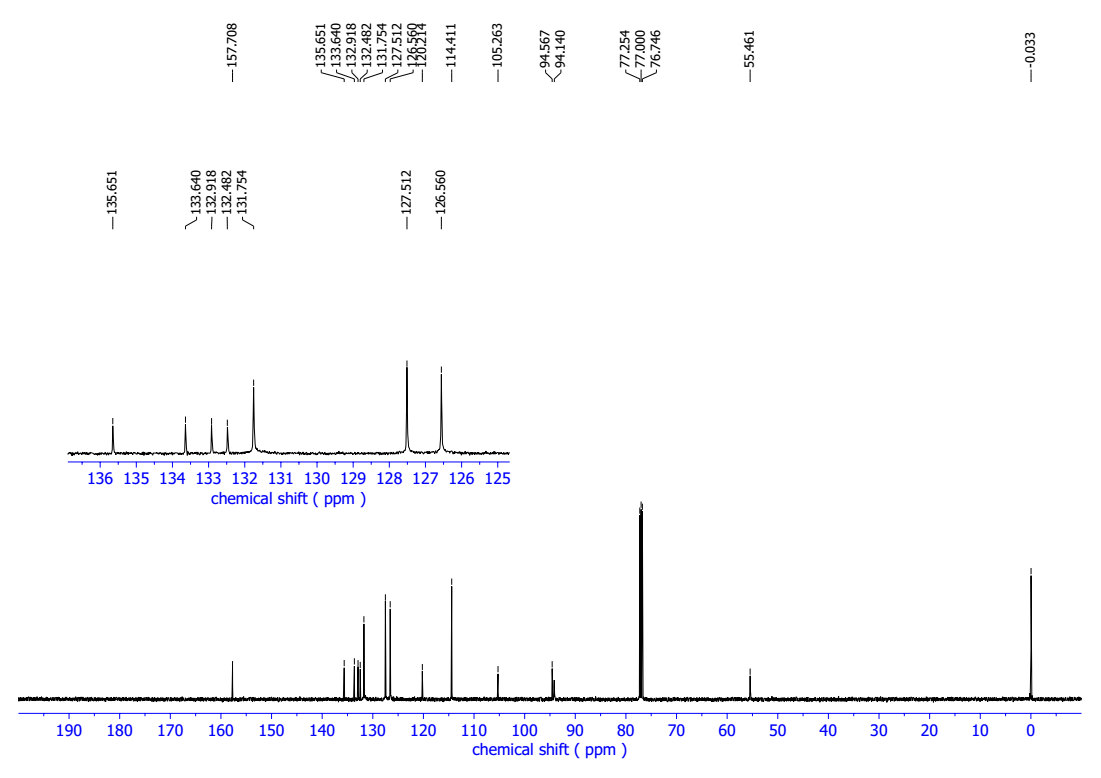
**

**Figure S6**. ^13^C{^1^H} NMR (125 MHz, CDCl_3_) Spectrum of TAPP **13.**

**
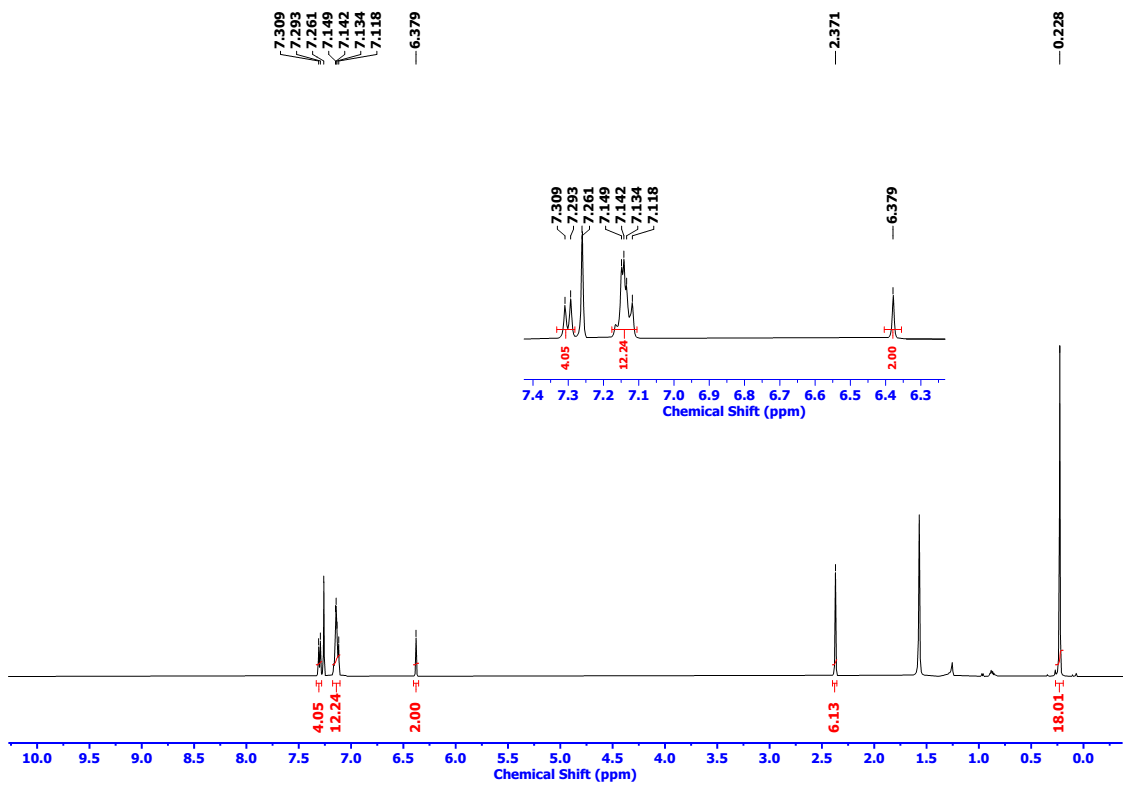
**

**Figure S7**. ^1^H NMR (500 MHz, CDCl_3_) Spectrum of TAPP **14.**


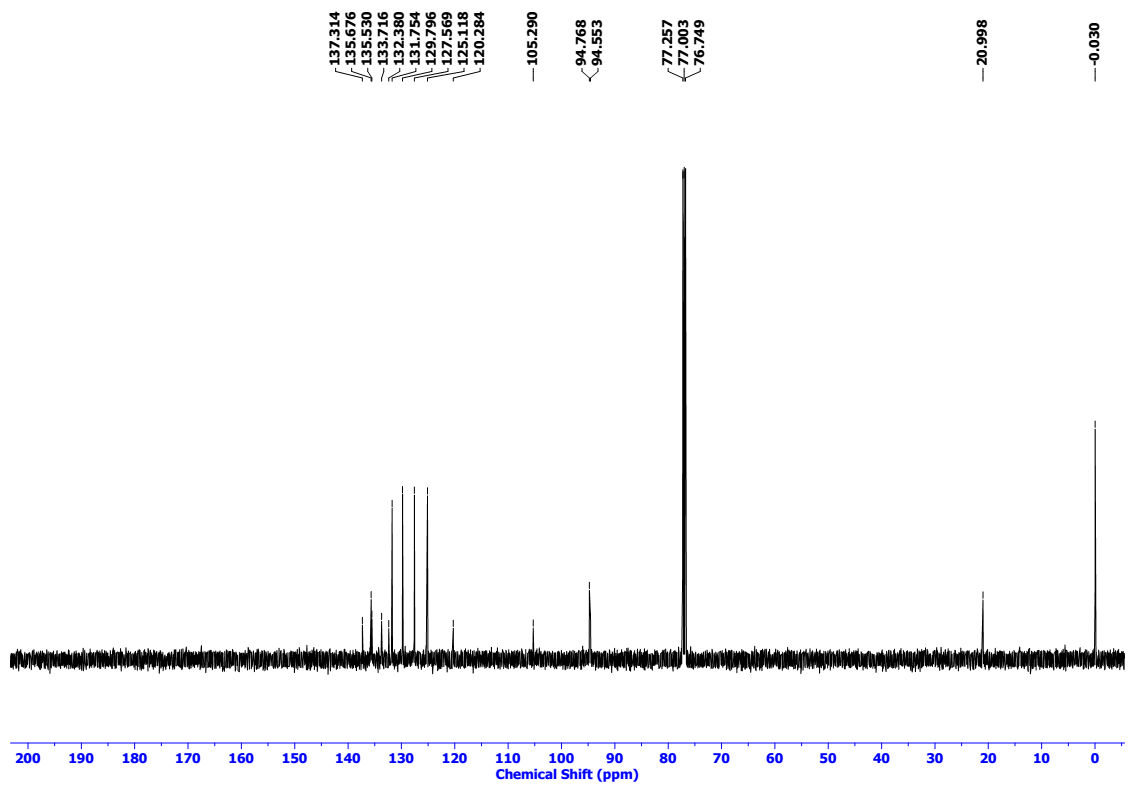


**Figure S8**. ^13^C{^1^H} NMR (125 MHz, CDCl_3_) Spectrum of TAPP **14.**

**
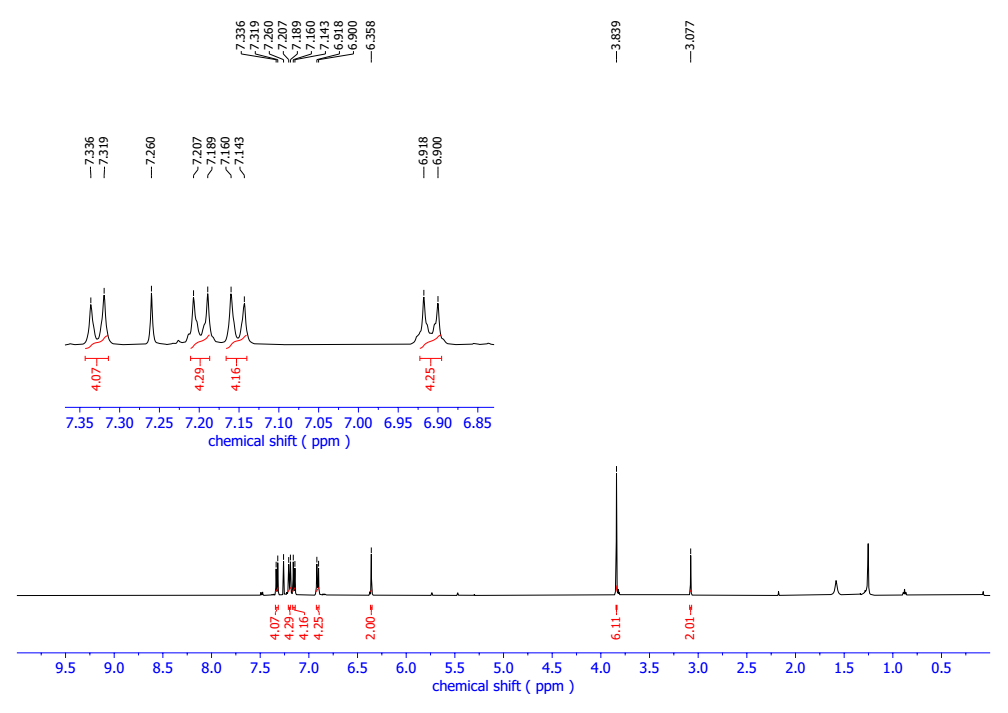
**

**Figure S9**. ^1^H NMR (500 MHz, CDCl_3_) Spectrum of TAPP **2.**

**
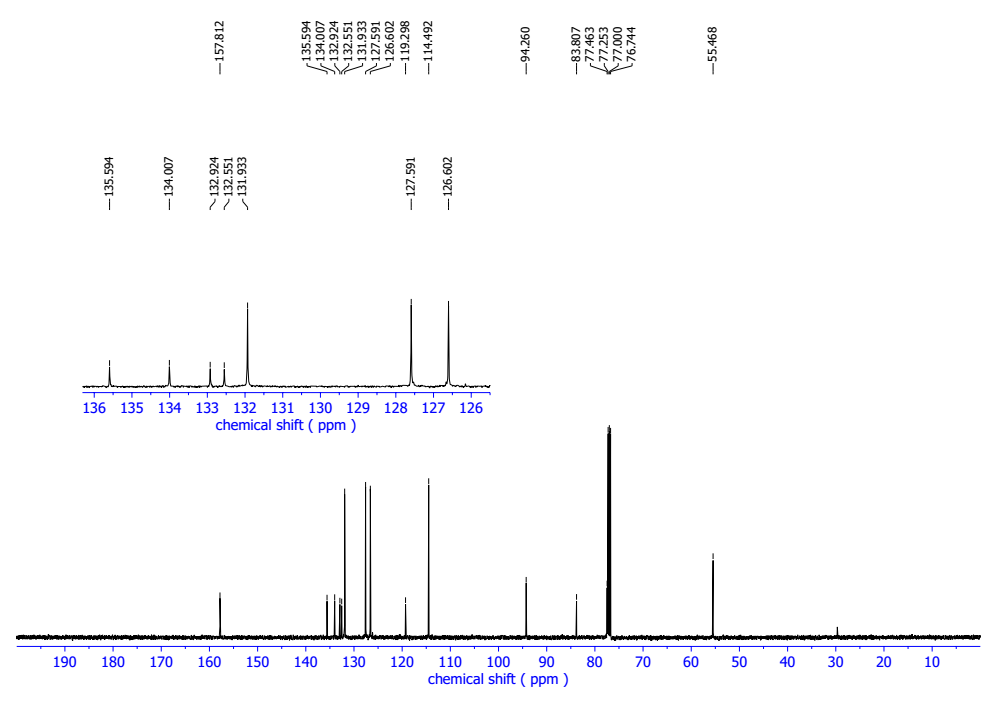
**

**Figure S10**. ^13^C{^1^H} NMR (125 MHz, CDCl_3_) Spectrum of TAPP **2.**

**
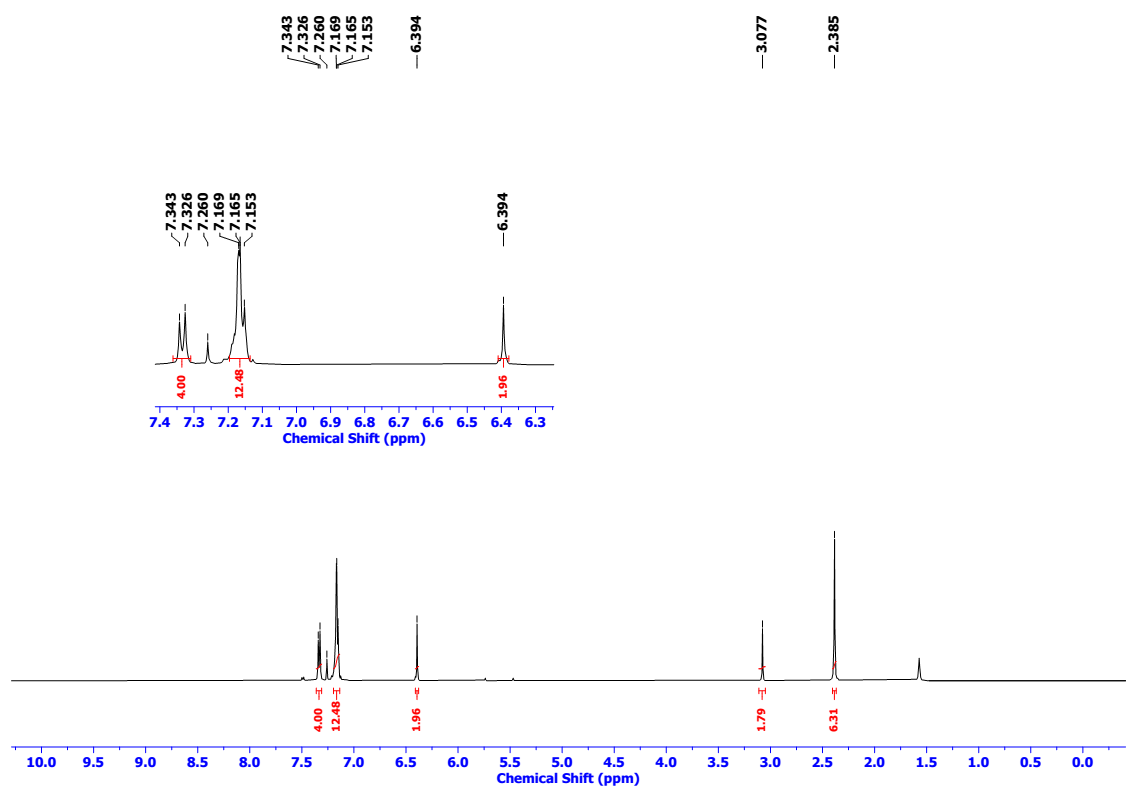
**

**Figure S11**. ^1^H NMR (500 MHz, CDCl_3_) Spectrum of TAPP **3.**

**
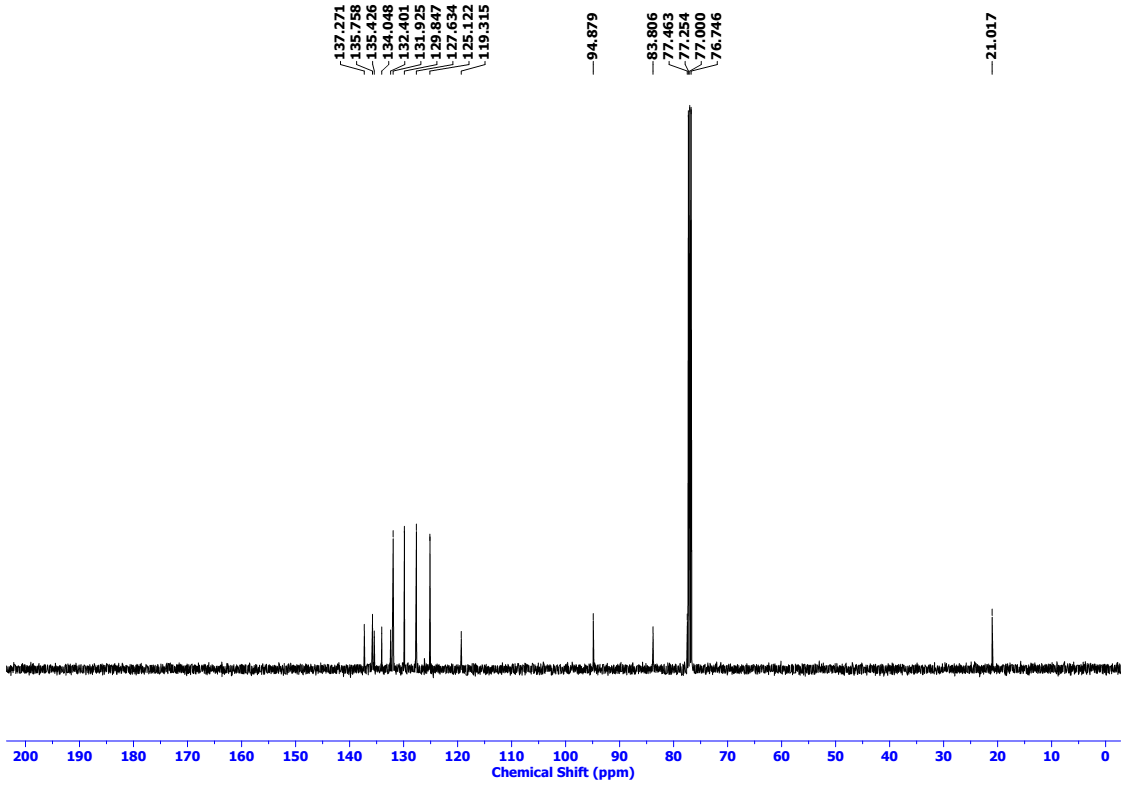
**

**Figure S12**. ^13^C{^1^H} NMR (125 MHz, CDCl_3_) Spectrum of TAPP **3.**

**
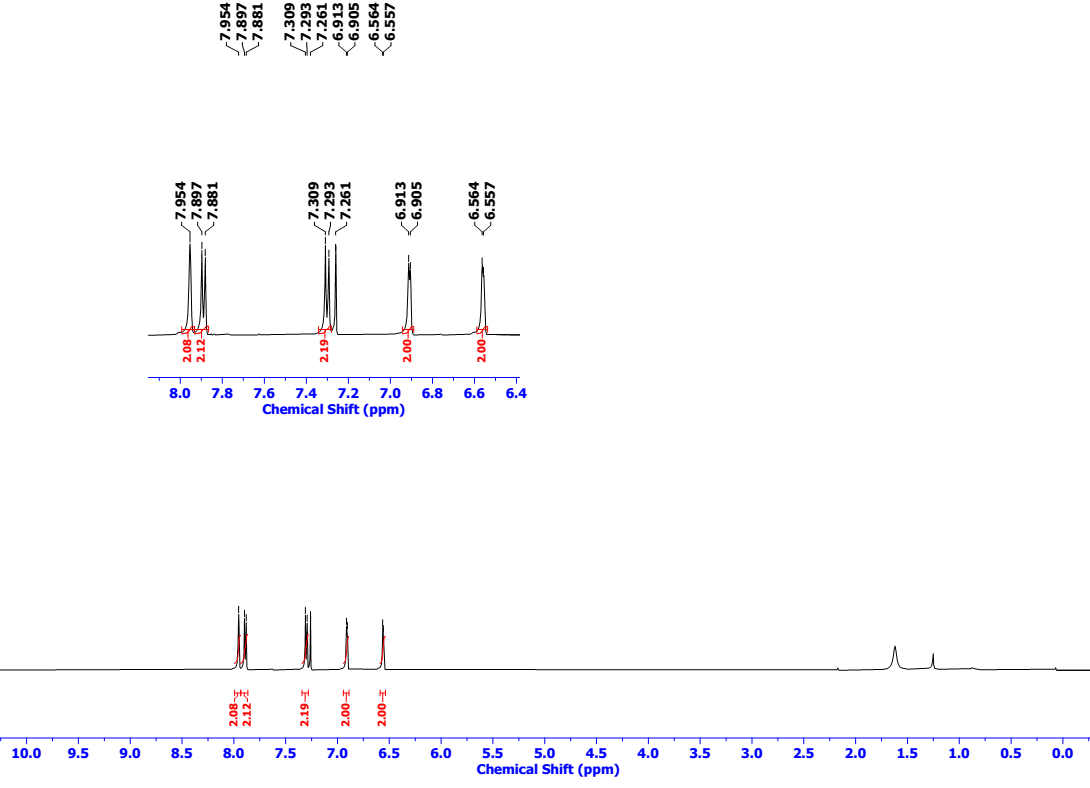
**

**Figure S13**. ^1^H NMR (500 MHz, CDCl_3_) Spectrum of BODIPY **9.**

**
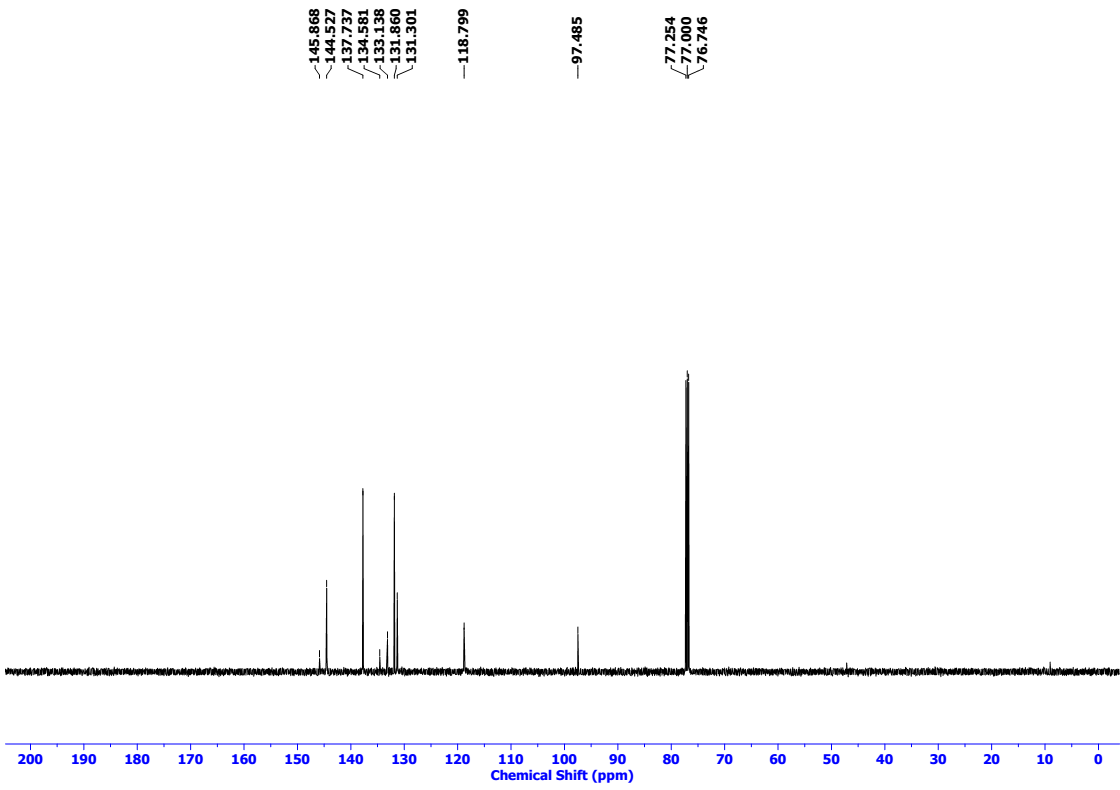
**

**Figure S14**. ^13^C{^1^H} NMR (125 MHz, CDCl_3_) Spectrum of BODIPY **9.**

**
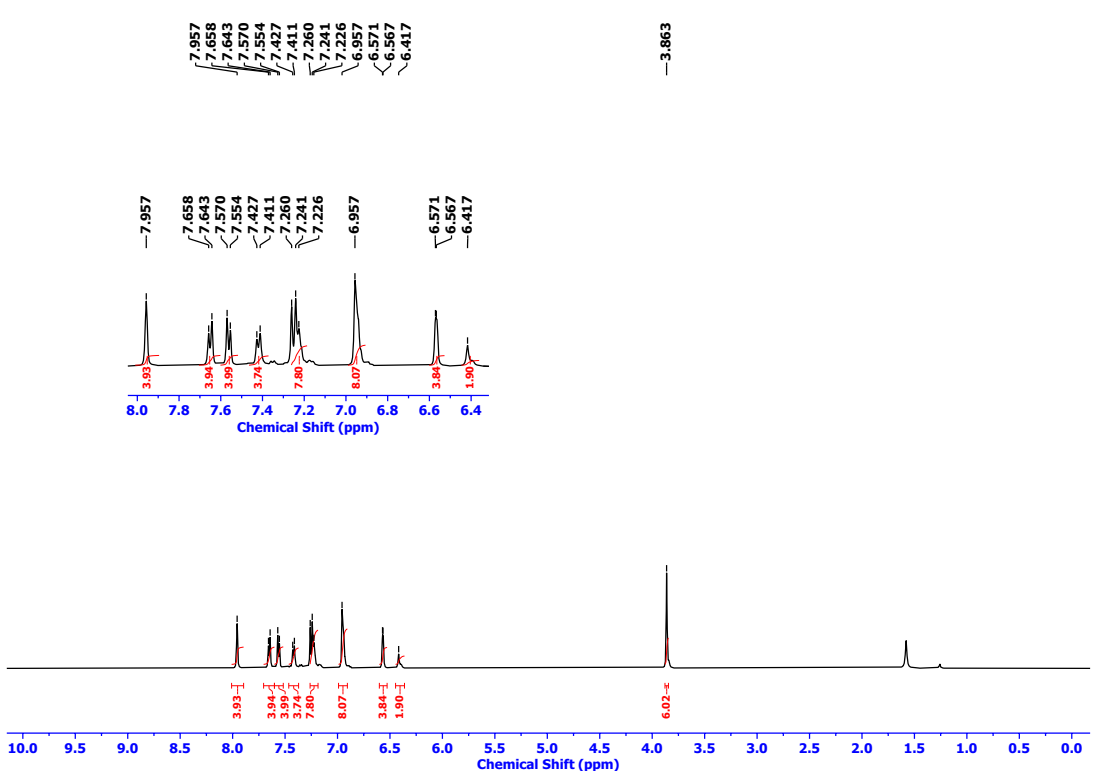
**

**Figure S15**. ^1^H NMR (500 MHz, CDCl_3_) Spectrum of Dyad **4.**

**
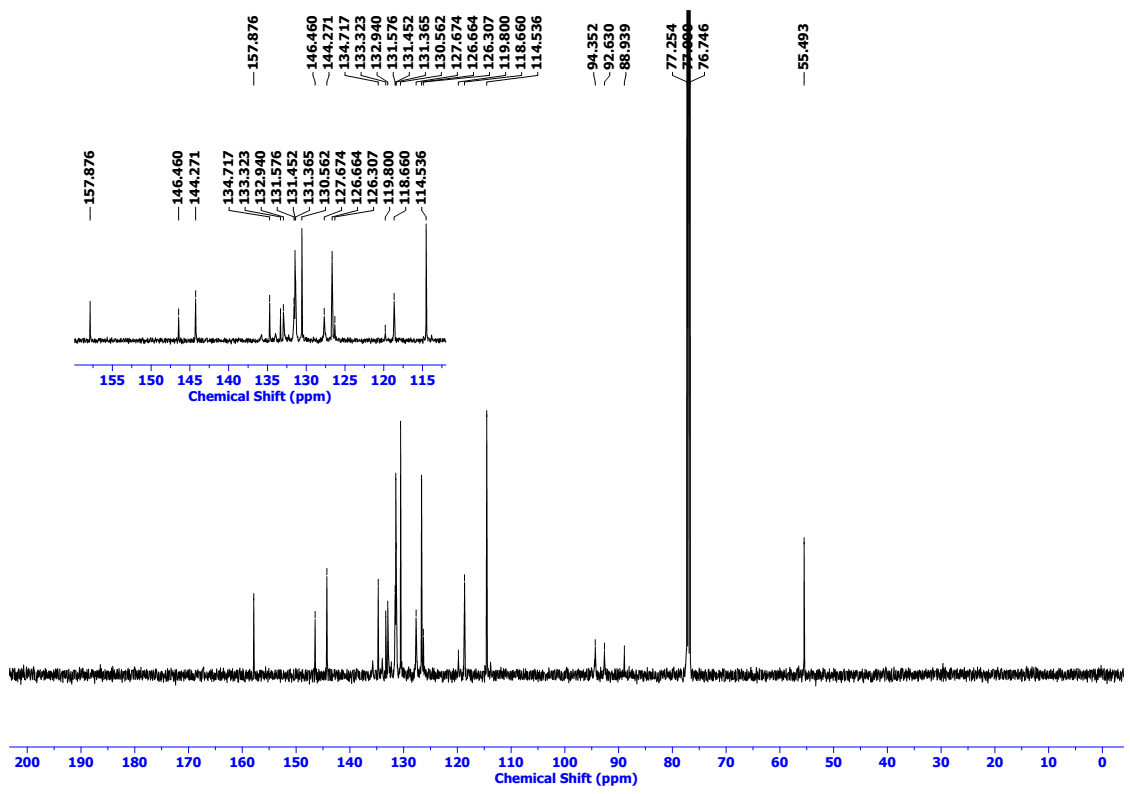
**

**Figure S16**. ^13^C{^1^H} NMR (125 MHz, CDCl_3_) Spectrum of Dyad **4.**

**
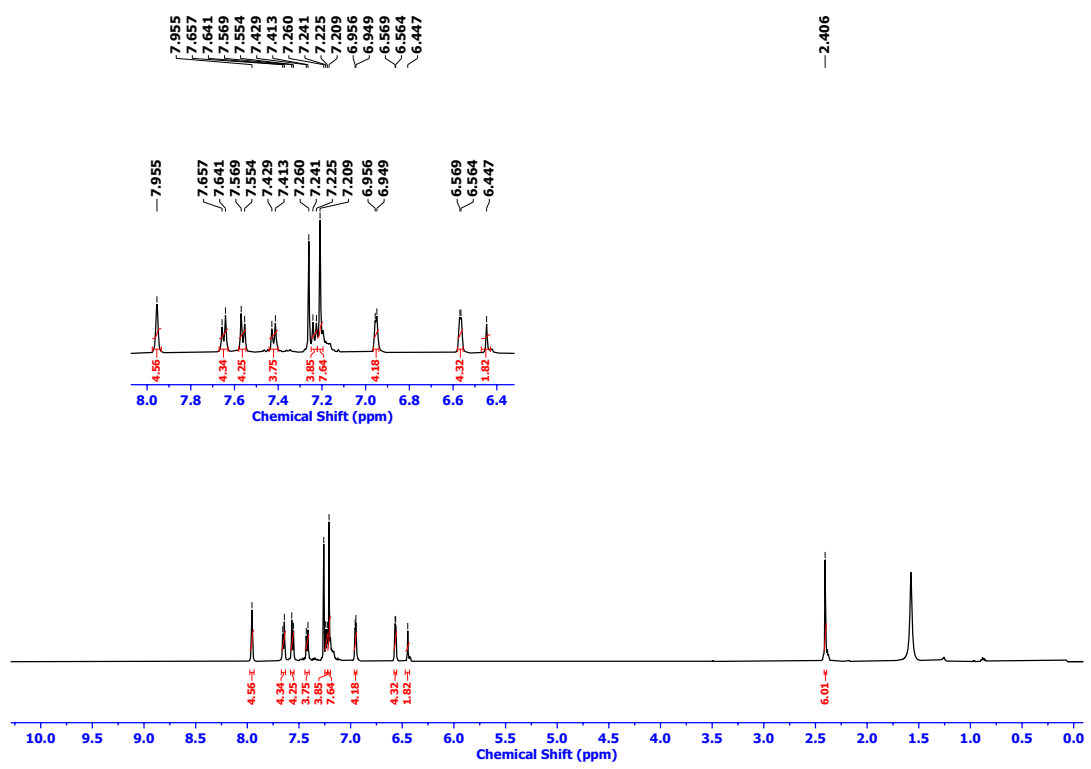
**

**Figure S17**. ^1^H NMR (500 MHz, CDCl_3_) Spectrum of Dyad **5.**

**
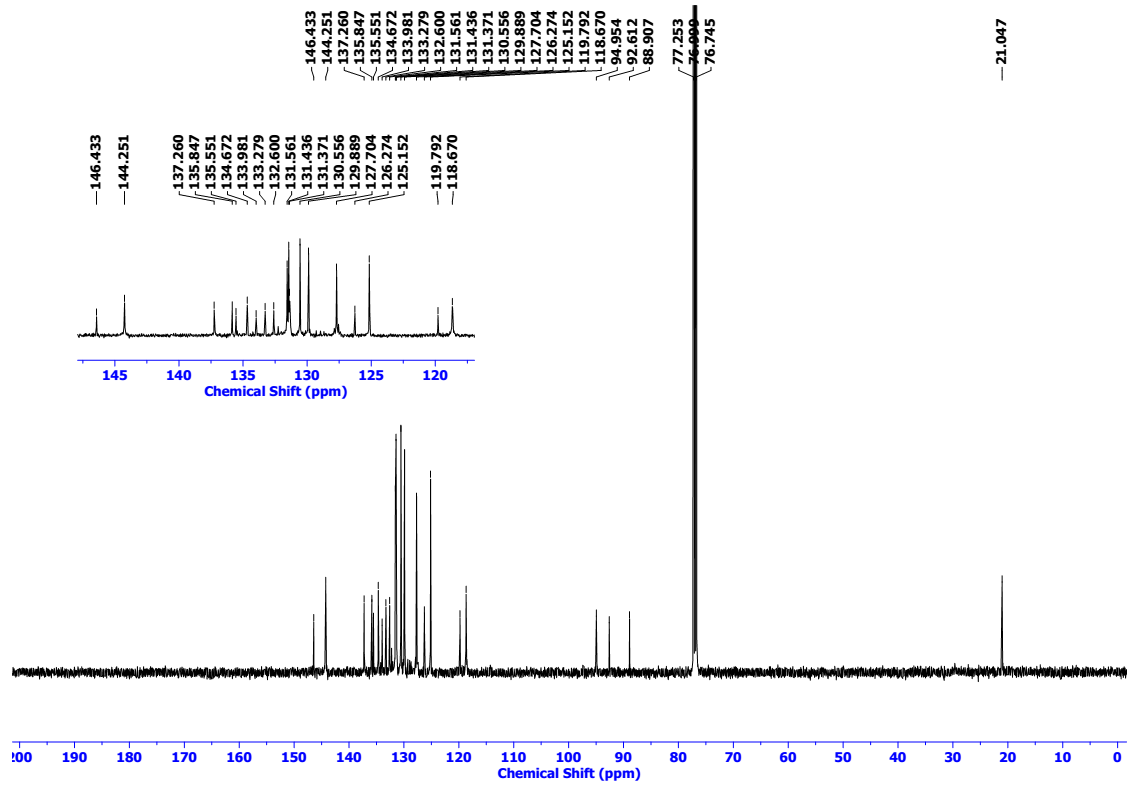
**

**Figure S18**. ^13^C{^1^H} NMR (125 MHz, CDCl_3_) Spectrum of Dyad **5.**

1. **References**

[1] CrysAlis PRO. Rigaku Oxford Diffraction, Yarnton, England, 2015.

[2] Sheldrick, G. M. (2015). SHELXT – Integrated space-group and crystal-structure determination. *Acta Cryst.* A71, 3–8, doi:10.1107/S2053273314026370.

[3] Dolomanov, O. V. Bourhis, L. J. Gildea, R. J. Howard, J. A. K. Puschmann, H. (2009). OLEX2 : a complete structure solution, refinement and analysis program. *J. Appl. Cryst.* 42, 339-341, doi:10.1107/S0021889808042726.

[4] Farrugia, L. J. (1997). ORTEP -3 for Windows - a version of ORTEP -III with a Graphical User Interface (GUI). *J. Appl. Crystallogr.* 30, 565–565. doi: 10.1107/S0021889897003117
